# Towards discovery of new leishmanicidal scaffolds able to inhibit *Leishmania* GSK-3

**DOI:** 10.1080/14756366.2019.1693704

**Published:** 2019-11-22

**Authors:** Paula Martínez de Iturrate, Victor Sebastián-Pérez, Montserrat Nácher-Vázquez, Catherine S. Tremper, Despina Smirlis, Julio Martín, Ana Martínez, Nuria E. Campillo, Luis Rivas, Carmen Gil

**Affiliations:** a Centro de Investigaciones Biológicas (CIB-CSIC), Madrid, Spain; b Microbiology Department, Hellenic Pasteur Institute, Athens, Greece; c Global Health R&D, GlaxoSmithKline, Tres Cantos, Spain

**Keywords:** *Leishmania*, GSK-3, Leishbox, molecular modelling

## Abstract

Previous reports have validated the glycogen synthase kinase-3 (GSK-3) as a druggable target against the human protozoan parasite *Leishmania*. This prompted us to search for new leishmanicidal scaffolds as inhibitors of this enzyme from our in-house library of human GSK-3β inhibitors, as well as from the Leishbox collection of leishmanicidal compounds developed by GlaxoSmithKline. As a result, new leishmanicidal inhibitors acting on *Leishmania* GSK-3 at micromolar concentrations were found. These inhibitors belong to six different chemical classes (thiadiazolidindione, halomethylketone, maleimide, benzoimidazole, *N*-phenylpyrimidine-2-amine and oxadiazole). In addition, the binding mode of the most active compounds into *Leishmania* GSK-3 was approached using computational tools. On the whole, we have uncovered new chemical scaffolds with an appealing prospective in the development and use of *Leishmania* GSK-3 inhibitors against this infectious protozoan.

## Graphical Abstract

**Figure:**
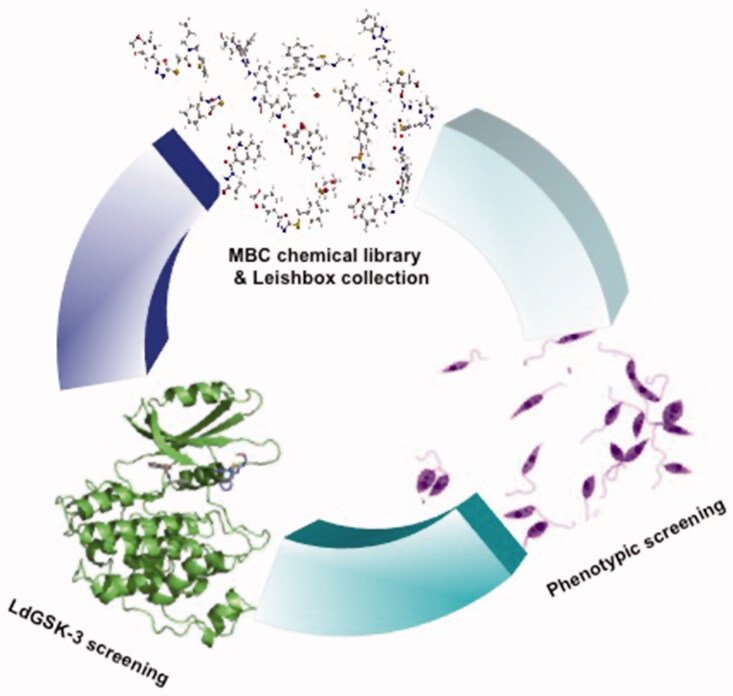

## Introduction

Human leishmaniasis is caused by infection with 20 protozoan species belonging to the genus *Leishmania*. Between 10 and 12 million people are infected worldwide, with 1–2 new million cases per year, and 20,000–50,000 deaths per year^1^.

Nowadays, leishmaniasis treatment is almost exclusively limited to chemotherapy and confined to four main drugs: organic antimonials, amphotericin B, miltefosine and paromomycin. Moreover, the current effectiveness of the available therapeutic options is increasingly eroded by rising resistance, implementation costs in low income endemic countries, and lack of treatment compliance^2^. Furthermore, the number of new drugs under clinical assays is rather scarce, and new alternatives in terms of drugs and validated targets are needed to avert this gloomy landscape^3^.

Remarkably, modulation of protein kinase (PK) activity is an appealing pharmacological approach to be exploited as a drug target, as PKs regulate a variety of essential processes in every living organism. Nowadays, the druggable kinome is the largest group in pharmacology^4^. Thirty-seven human protein kinase inhibitors (PKi) have been approved for clinical use and more than two hundred additional PKis are under clinical trial^5^. The aim of these PKis is to correct aberrant protein phosphorylation syndromes underlying human pathologies such as, but not limited to, cancer or neurodegenerative diseases^6^^,^^7^.

The characterisation of the divergences between the kinome of several parasites from those of their mammalian host has become a source of new targets for novel anti-infective drugs^8^^,^^9^. Moreover, the pharmacological role of PKis has been extended beyond the selective inhibition of PKs from the pathogen to revert changes in the protein phosphorylation pattern of the host, driven by the pathogen in order to ensure its survival inside the host^10^^,^^11^. Concerning the former mode of action, two different approaches have been used: first, the design of PKis for atypical kinases of the pathogen^12^ which are absent in the host, and second, to exploit the often subtle differences between homologue kinases of the parasite and of the host^13^.

The *Leishmania major* genome contains 199 PKs^14^, exclusively belonging to the Ser/Thr kinases, except for some kinases with dual activity as Ser/Thr or Tyr kinases. Protein kinases are involved in processes such as the inter-stage differentiation during the life cycle of the parasite^15–17^, macrophage invasion^18^, response to stress^19^, intracellular survival in the host^20^^,^^21^, or drug resistance^22^, among others. Several groups have provided a consistent proof of concept on the druggability of *Leishmania* kinome as a new chemotherapy venue^23^^,^^24^. In fact, protein phosphorylation site descriptors for *Leishmania* proteins have been reported^25^, and PK inhibition was carried out for a number of enzymes, such as Akt-like^26^, CK1.2^27^, PKA^28^, PKC^29^^,^^30^, Aurora kinase^31^, as well as GSK-3^32–34^.

In mammalian cells, glycogen synthase kinase-3 (GSK-3) is a multitask Ser/Thr kinase with functionalities far beyond its inceptive regulation of the last step in glycogen biosynthesis. GSK-3 is involved in a number of signalling pathways implicated in the regulation of processes such as receptor signalling, cell proliferation, cell differentiation and death, embryonic development, glycogen and energy metabolism^35^, all accounting for its druggability in human pathologies.

Counterparts of human GSK-3 have been described in *Leishmania spp.* and *Trypanosoma brucei*. Both organisms express short- and long-forms of the enzyme, each of them encoded by different genes. Inhibition of GSK-3 expression by RNAi in the bloodstream form of *Trypanosoma brucei* evidenced the greater importance of short GSK-3 over its long isoform for parasite viability^32^. Both forms have the same active site, hence it can be surmised that their pharmacological inhibition is likely to be similar^34^. There is a 41% identity in amino acids between the short form of *Leishmania major* GSK-3 (LmjGSK-3) and the GSK-3β in humans^34^. Nevertheless, these differences are reduced to just 3 of the 21 amino acid sequence lining the active site.

*Leishmania donovani* GSK-3 (LdGSK-3) was inhibited by 6-bromo-5-methylindirubin-3'-oxime causing cell cycle deregulation and induction of the apoptosis. These lethal effects were partially rescued by overexpression of the short form of LdGSK-3, hence the enzyme was genetically and pharmacologically validated^33^. The sequence of the short form of GSK-3 was preserved in *L. donovani*, *L. infantum,* and *L. major*^36^, thus, inhibitors against one form of the disease, either visceral or cutaneous, will presumably be active on the other forms, enticing their potentiality. Moreover, the anti-inflammatory effects of human GSK-3β (hGSK-3β) inhibitors may reduce the inflammatory pathology associated with leishmaniasis^37^.

These facts prompted us to search for novel leishmanicidal scaffolds where the inhibition of *Leishmania* GSK-3 played a crucial role. For this purpose, a dual yet complementary approach was taken to enlarge the chemical space explored. First, we evaluated a small set of hGSK-3β inhibitors previously developed in our group, chemically diverse and with different binding modes to the human enzyme. These inhibitors were assayed as leishmanicidal agents and complemented with the *in vitro* assessment of the inhibition of short LdGSK-3. Next, we explored 186 compounds from the Leishbox collection as LdGSK-3 inhibitors. Leishbox belongs to TCAKS (i.e., Tres Cantos Anti-Kinetoplastids Set, aka Kinetoboxes), an open access collection of compounds selected through the screening of 1.8 million molecules from the GlaxoSmithKline chemical library for leishmanicidal and trypanocidal activity particularly enriched with chemotypes described as putative protein kinase inhibitors, according to a bioinformatic analysis^38^. Compounds in Leishbox are active against axenic and intracellular *L. donovani* amastigotes (*IC*_50_ ≤5 µM), and devoid of cytotoxicity on HepG2 and THP-1 cells.

On the whole, we have uncovered 11 new inhibitors for GSK-3 with leishmanicidal activity belonging to 6 different chemical classes, with activities at the low micromolar range. The molecular basis of their inhibition was predicted by molecular docking studies. Furthermore, new chemical scaffolds have been added to the list of active leishmanicidal compounds with GSK-3 inhibitory properties to be further optimised.

## Material and methods

### Reagents

All the commercial reagents were of the highest quality available. Unless otherwise stated, they were purchased from SIGMA-Aldrich Spain. RPMI 1640 medium, RPMI 1640 without red phenol, and M199 medium, were obtained from Gibco.

### Compounds studied

Human kinase inhibitors tested in this work (Table S1) were previously described^39–47^ and are collected in the MBC chemical library^48^. Their purity was determined by elemental analysis and values were within ±0.4% of the theoretical values. Leishbox compounds^38^ (Table S2) were kindly provided by GlaxoSmithKline as 10 µL of 10 mM solution in DMSO.

### Cells

*Leishmania donovani* promastigotes (MHOM/SD/00/1S-2D) were grown at 26 °C in RPMI 1640 medium supplemented with 5 mM HEPES, 1.7 mM HCO_3_Na, 10% HIFCS (Heat-Inactivated Foetal Calf Serum; Biowest), 2 mM L-glutamine, 20 U/mL unicillin (ERN Laboratories, S.A.), 24 µg/mL gentamicin (NORMON Laboratories, S.A.); pH 6.8–6.9 (RPMI 1640-HIFCS). *L. pifanoi* axenic amastigotes (MHOM/VE/60/Ltrod) were grown at 32 °C in M199 medium supplemented with 20% HIFCS, 0.5% trypticase peptone (BD Biosciences), 13.9 mM D-glucose, 76.7 µM haemin, 5.1 mM glutamine, 40 µg/mL gentamicin; pH 7–7.2 (M199-HIFCS).

### Buffers

Kinase PBS (150 mM NaCl, 1.5 mM H_2_KPO_4_, 2.7 mM KCl, 8.3 mM HNa_2_PO_4_, 60 mM β-glycerophosphate disodium salt, 1 mM Na_3_VO_4_, 1 mM NaF, 1 mM disodium phenyl phosphate; pH 7.5); Lysis Buffer (Kinase PBS plus 10 mM imidazole and protease inhibitors cocktail (Roche Ref. 1697498); pH 7.5), Washing Buffer (Kinase PBS with NaCl at 300 mM, plus 30 mM imidazole, 1% Triton X-100 and protease inhibitors cocktail; pH 7.5), Elution Buffer (Kinase PBS plus 300 mM imidazole and protease inhibitors cocktail; pH 7.5) and Kinase Assay Buffer (50 mM HEPES pH 7.5, 1 mM EGTA, 1 mM EDTA, 15 mM Mg(AcO)_2_, 0.1 mg/mL bovine seroalbumin).

### Cell harvesting

*Leishmania* parasites were collected at late exponential growth phase by centrifugation at 1610×*g* at 4 °C. Peritoneal murine macrophages were obtained from 8-week-old Balb/c mice previously elicited by i.p. injection with 1 mL of 10% thioglycollate medium three days prior to extraction. Macrophages were obtained by peritoneal washing (10 mL PBS, 4 °C). After extraction, macrophages were maintained in RPMI 1640-HIFCS at 37 °C and 5% CO_2_. All the animal procedures were approved by the welfare animal ethical Committee of the CSIC and the Autonomous Government of Madrid (authorization number: PROEX 070/18) and followed the 3 R principle.

### Leishmanicidal and cytotoxicity assays

*Leishmania* parasites were resuspended with the corresponding drug concentration at 2 × 10^6^ parasites/mL (final concentration) in their respective growth media in 96 microwell plates (200 µL/well). The parasites were allowed to growth for 72 h at 26 °C for promastigotes and 96 h at 32 °C for axenic amastigotes. Afterwards, inhibition of proliferation was measured by the inhibition of MTT reduction by the parasites. To this end, MTT (3–(4,5-dimethylthiazol-2-yl)-2,5-diphenyltetrazolium bromide) was added to each well (0.5 mg/mL, final concentration). MTT reduction was allowed to proceed for 2 h at the respective temperature, and the resulting formazan solubilised by addition of 50 µL/well of 10% SDS. Afterwards, the plate was read at 595 nm in a BioRad Microplate-reader, model 680.

For peritoneal macrophages, the cells were seeded in 96 microwell plates at 1 × 10^5^ cells/well and incubated (37 °C, 5% CO_2_) with the drug for 48 h. The toxicity was evaluated by the inhibition of MTT reduction as above.

Samples were made by triplicate and assays were repeated at least twice. EC_50_ (effective concentration capable that inhibits parasite growth by 50%) was calculated using the statistical module of SigmaPlot v11.0 software.

### Gene cloning

The *LdGSK-3s* gene was obtained from pTriEx-1.1-LdGSK-3s expression vector^33^ and cloned into the plasmid pET28 a(+) (Novagene) by digestion with NcoI and XhoI (New England Biolabs) and further ligation with the T4 DNA ligase (New England Biolabs) to obtain the pET28a(+)-*Ld*GSK-3s recombinant plasmid. *E. coli* BL21(DE3) was electroporated (25 µF, 2.5 kV, 200 Ω, 0.2 cm cuvettes) with this plasmid. Transformants were incubated (2 h, 37 °C) in LB medium supplemented with 10 mM MgCl_2_, 10 mM MgSO_4_ and 20 mM D-glucose and further selected in LB agar plates supplemented with kanamycin at 50 µg/mL. The new plasmid construct was confirmed by automated sequencing.

### Extraction and purification of recombinant LdGSK-3

Expression of LdGSK-3 in *E. coli* BL21(DE3) (pET-28a(+)-*Ld*GSK-3, O.D._595nm_= 0.6) was induced with 1 mM IPTG (3 h at 37 °C) in selected kanamycin LB media in agitation. Culture was centrifuged at 10,800×*g* (30 min, 4 °C). Afterwards the pellet was resuspended in Lysis Buffer, and disrupted in a French Press (Thermo Scientific). Then, 0.1% Triton X-100 (final concentration) was added to the lysate, and further incubated for 30 min at 4 °C with shaking. The non-soluble material was removed by centrifugation (38,730×*g*, 30 min, 4 °C). The supernatant was loaded into an equilibrated Ni-column (HisTrap^TM^ FF crude 5 mL, Merck, Ref. 17–5286-01). The non-bound material was removed by Washing Buffer and LdGSK-3 was obtained from the column using the Elution Buffer. The resulting fractions were kept at –80 °C in 15% glycerol. GSK-3 activity was measured using the Kinase Glo^TM^ protocol described below. Fractions and samples from the different purification steps were analysed for kinase activity *(vide infra)*, protein content evaluated by Bradford reagent (Bio-Rad), and their protein pattern obtained by SDS-PAGE electrophoresis on a 10% polyacrylamide gel. Fractions with the highest GSK-3 activity were pooled and dialysed using an Amicon cell filtration (30 kDa cut-off membrane) using Kinase PBS as washing buffer. LdGSK-3 was quantified after ultracentrifugation (147,000×*g*, 1 h, 4 °C), filtrated through a 0.22 µm nitrocellulose filter and preserved at −80 °C.

### Assessment of inhibition of LdGSK-3 with Kinase Glo^TM^

Compounds were tested against 20 ng of purified LdGSK-3 in the presence of 25 µM Phosphoglycogen Synthase Peptide-2 (Millipore, Ref. 12–241) and 1 µM ATP (Sigma-Aldrich, Ref. A7699) in Kinase Assay Buffer in 96-well black plates. The reaction was allowed to proceed for 30 min at 30 °C, then it was incubated at room temperature for another 10 min after addition of Kinase Glo reagents (Promega, Ref. V6712). ATP consumption by LdGSK-3 was measured indirectly through end-point luminescence measurement of each well in a Varioskan Flash microplate reader (Thermo).

### Sequence analysis and alignments

All available sequences in the Uniprot database^49^ corresponding to *Leishmania spp*. GSK-3 and human GSK-3 were retrieved. First, as the only GSK-3 crystal structure available in *Leishmania* is from *L. major,* this sequence was compared with that from *L. donovani*, and further extended to the rest of GSK-3 sequences available for *Leishmania*. In all cases, sequence alignments were performed using Clustal omega software and default parameters were selected^50^ (Figures S1 and S2).

### Ligand preparation

For the development of this work, all the compounds used were prepared and converted into 3 D structures for computational studies applying Ligprep tool^51^, a module of the Schrödinger software package. The most reliable ionisation states of the compounds were optimised at pH 7.3 as assigned by the Epik module. Finally, no tautomers were generated and all the compounds were desalted. During this process, the search was restricted to the compound with the low energy ring conformation and its most probable stereoisomer as found by the programme. As a final step, OPLS 2005 force field was applied in the energy minimisation process of the generated 3 D conformers^52^.

### Protein preparation

The crystal structure 3E3P of LmjGSK-3^34^ was retrieved from the PDB. However, in this crystallographic model the structure of the decapeptide located at the upper part of the ATP binding pocket was absent. This gap was modelled with the Modeller programe, and the whole protein was subsequently prepared using the Protein Preparation Wizard tool implemented on Maestro^53^^,^^54^. Hydrogens were added and the water molecules removed. All the residues were optimised according to their pKa and their corresponding protonation state as calculated at a physiological pH of 7.3.

### Docking studies

Automated docking was used to assess the appropriate binding orientations and conformations of the ligands. To this end, a Lamarckian genetic algorithm method of the AutoDock 4.2 programme was employed^55^^,^^56^. For docking calculations, Gasteiger charges were added to the ligands and to the protein, rotatable bonds were set by AutoDock tools (ADT), and all accepted torsions were allowed to the ligand. For the blind docking, a maximum grid of 126 × 126 × 126 points and a grid-point spacing of 0.375 Å was set up to include the whole surface of the protein. The macromolecule centre was selected as the centroid of the grid. When the docking was focussed in a specific pocket, grid maps with a grid box size of 50 × 50 × 50 points and a grid-point spacing of 0.375 Å were used. The docking protocol consisted of 200 independent Genetic Algorithm (GA) runs, population size of 150, a maximum evaluation number of 250,000 and default values for the remaining parameters.

For covalent docking, a special map for the attachment site of the covalent ligand was obtained by the grid-based approach. A Gaussian function is constructed with zero energy at the site of attachment, and steep energetic penalties at the surrounding areas. Docking analysis was performed using a specific atom type for the atom that forms the covalent linkage. Cys169 was selected as the centroid of the grid and its sulphur atom defined as the site of covalent binding.

In all cases, visual inspection was carried out for the final best docked clusters, defined as a < 2.0 Å RMSD default according to the binding energies and the relative population as provided by Autodock.

Induced Fit Docking (IFD)^57^^,^^58^ is based on fitting the ligand into the protein binding site allowing changes in the geometry of the nearby residues, mostly in the side chains. For this purpose, Prime^59^ predicts the active site structure and minimising the overall energy of the protein. Finally, each ligand is re-docked into its corresponding low energy protein structures and the resulting complexes rank according to docking score. XP (extra precision) mode was used in a standard protocol and no constraints were set, residues were optimised to 5.0 Å of the ligand poses while the rest of the parameters were set as default. The best structures were selected to carry out further docking studies.

## Results and discussion

### hGSK-3β inhibitors as a source of LdGSK-3 inhibitors

An enzymatic and phenotypic screening of a selected set of 24 hGSK-3β inhibitors was carried out in order to find potential drugs for leishmaniasis. These heterocyclic inhibitors were designed and synthesised in our laboratory and chosen according to their chemical diversity and binding mode to hGSK-3β enzyme as previously reported. The selection included thiadiazole, thiophene, quinoline, thiazole and maleimide derivatives, acting as ATP-non competitive^39–41^, substrate competitive^42^, allosteric^43^^,^^44^, and reversible or irreversible ATP-competitive^45^ inhibitors. Notably, one of the inhibitors tested, tideglusib (**2**), is under current clinical trials for neurological disorders^60^.

Compounds **1–24** were simultaneously evaluated against the *in vitro* inhibition of LdGSK-3 as well as in phenotypic assays for leishmanicidal activities (Table S1). The enzyme was expressed and purified based on a previous protocol with subtle modifications^33^, and the enzymatic activity measured by luminescence evaluation of the remaining ATP. Moreover, compounds were tested in *Leishmania* cultures (*L. infantum* promastigotes and *L. pifanoi* axenic amastigotes), and their cytotoxicity evaluated in peritoneal murine macrophages (PMM).

After these initial studies, eleven showed a good inhibition of LdGSK-3 and some of them also phenotypic activities. From the initial set, eight were selected as representative members of the different chemical families according to their binding modes to the human enzyme as well as to their biological activities. As shown in Table 1, all the selected compounds inhibited LdGSK-3, except quinoline **11** and maleimide **13**. Thiadiazolidinones (TDZDs) **1** and **2**, and halomethylketones (HMKs) **5** and **14**, inhibited LdGSK-3, and also showed a higher leishmanicidal activity on axenic amastigotes than on promastigotes. Compounds **3** and **4**, both 5-imino-1,2,4-thiadiazoles (ITDZs), showed nil antiparasitic activities, despite their significant *in vitro* inhibition of the enzyme.

**Table 1.** *In vitro* enzymatic and antiparasitic activities of selected hGSK-3β inhibitors.^a^

Compound	Chemical structure	hGSK-3β *IC*_50_ (μM)	LdGSK-3^b^ *IC*_50_ (μM)	*L. infantum* promastigotes EC_50_ (μM)	*L. pifanoi* amast. ax EC_50_ (μM)	PMM^c^ EC_50_ (μM)	SI^d^
**1**		2^39^	1.1 ± 0.2	10.9 ± 0.4	2.0 ± 1.9	32.9 ± 3.5	16.5
**2**		0.005^61^	0.32 ± 0.05	17.6 ± 2.3	7.1 ± 1.8	>50	>7.0
**3**		0.9 ± 0.1^42^	0.24 ± 0.01	>25	>50	–	–
**4**		2.0 ± 0.4^42^	0.17 ± 0.00	>25	>50	–	–
**5**		0.5^40^	1.8 ± 0.3	4.6 ± 0.2	2.2 ± 0.6	6.3 ± 1.2	2.9
**11**		3.01 ± 0.14^43^	<20%@10 μM	>50	3.6 ± 1.3	9.9 ± 0.9	2.8
**13**		0.89 ± 0.19^45^	<20%@10 μM	>50	>25	–	–
**14**		0.005 ± 0.001^45^	1.6 ± 0.2	>50	6.5 ± 2.0	>25	>3.8

^a^ *IC*_50_: 50% inhibitory concentration; EC_50_: 50% effective concentration.

^b^ Indirubin-3’-monoxime-5-sulphonic acid was used as reference of the assay: *IC*_50_ (LdGSK-3)= 2.4 ± 0.2 μM.

^c^ PMM: peritoneal murine macrophages.

^d^ SI: Specificity Index (EC_50_ PMM/EC_50_ amas. ax).

Compounds **1** and **2** are well-known members of the non-ATP competitive TDZD family of hGSK-3β inhibitors^39^ acting as covalent inhibitors through the formation of a disulphide bond with Cys199^61^. Since this Cys residue is conserved at the entrance of the ATP binding site of LdGSK-3 (Cys169), it is expected that TDZDs bind the parasitic enzyme through this residue. Moreover, HMKs **5** and **14** irreversibly inhibit the human enzyme forming a covalent bond with Cys199^41^. Similarly, **5** and **14** may bind LdGSK-3 in the same way. Two substrate competitive inhibitors of hGSK-3β, **3** and **4**, belonging to the ITDZ family^42^, were also active against LdGSK-3. The substrate binding pocket of the enzymes is quite conserved respect to the human one, so it is inferred that the ITDZs were substrate-competitive inhibitors of the *Leishmania* enzyme. Remarkably, the two ITDZs **3** and **4** inhibited LdGSK-3 at submicromolar concentrations, but were devoid of significant leishmanicidal activities, even at the highest concentration tested (50 µM). Likely they were unable to build-up an intracellular inhibitory concentration on the parasite, hence a strategy of conjugation with cell penetrating peptide-based (CPPs) is currently being pursued to overcome this drawback in order to improve the permeability of ITDZs^62^.

To account for the experimental results of the tested compounds and to confirm the hypothesis described above, molecular docking was carried out and compared with its human counterpart. The computational studies were based on the crystal structure of LmjGSK-3 (PDB code 3E3P)^34^. However, as the crystal structure of the protein lacks a decapeptide loop at the upper part of the ATP binding pocket, its sequence was modelled using the Modeller programme before the onset of the analysis^63^. Once the protein structure was completed, a minimisation step was further performed. As the crystal structure was in an apo form, the holo structure was mimicked by induced fit docking (IFD). Compound 6-bromo-5-methylindirubin-3′oxime (5-Me-6-BIO), one of the most potent ATP-competitive inhibitor for this enzyme^33^, was selected for this optimisation step.

**Figure 1.**
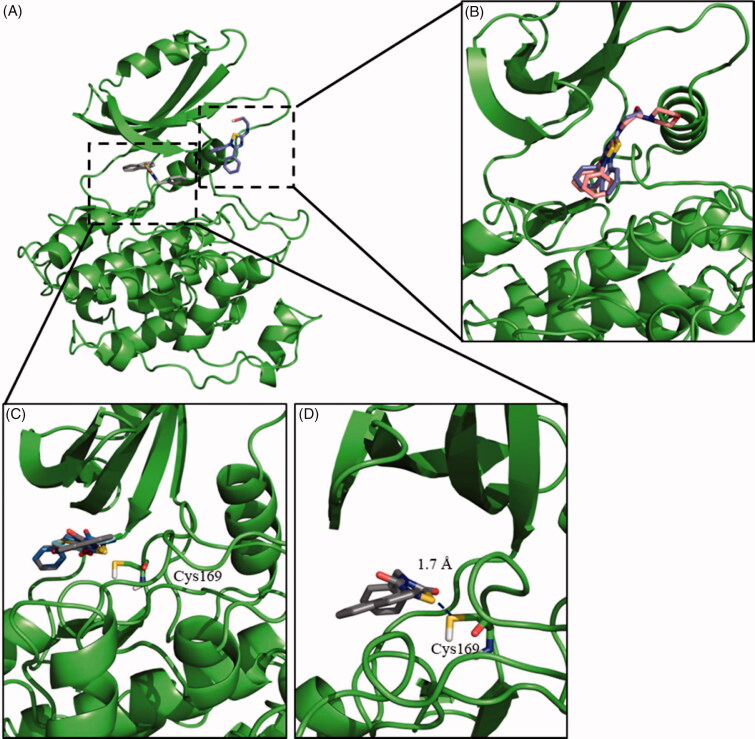
Binding mode into the LmjGSK-3 enzyme for ITDZ, TDZD, and HMK representative compounds. (A) Blind docking poses obtained from the most representative clusters for **2** (TDZD) and **4** (ITDZ) in the ATP binding site and the substrate binding site, respectively. (B) Superimposition of most representative regular docking results of ITDZ compounds **3** and **4**. (C) Superimposition of the best covalent docking poses obtained for TDZDs (**1** and **2**) and HMK **5**. (D) Detailed view of the covalent docking for compound **2**.

Regarding TDZD, ITDZ and HMK families, blind docking studies were carried out with compounds **2**, **4**, and **5** as representatives of the families, respectively. The results of the blind docking agreed with our hypothesis; TDZD **2** and HMKs **5** poses are allocated at the entrance of the ATP binding site, while for ITDZ **4** all the poses of a predominant cluster supported its binding in the substrate pocket. Therefore, in every case the binding mode for each of the families was confirmed, as illustrated in Figure 1(A). Based on these results, a more detailed study was carried out using additional regular docking study. For this, compounds **2** and **5** were selected as representative compounds for TDZDs and HMKs chemical families, as well as **4** (as representative of ITDZ family). This docking study was focussed on their previous predicted binding sites. The results confirmed a similar binding mode of the two ITDZ derivatives (**3** and **4**) inside the substrate binding pocket (Figure 1(B)). An important number of the poses of TDZs and HMK provided 3–4 Å distances between the reactive atoms of the inhibitors and the Cys169 of GSK-3 compatible with the formation of covalent bond. In order to validate this hypothesis, covalent docking for TDZDs (**1** and **2**) and HMK **5** was performed. Distances between 1.5–2.5 Å were obtained in most of the poses for compounds **1**, **2** and **5**, typical of a covalent bond (Figures 1(C,D)). In all, the data purported a covalent inhibition of the enzyme for the three compounds.

**Figure 2.**
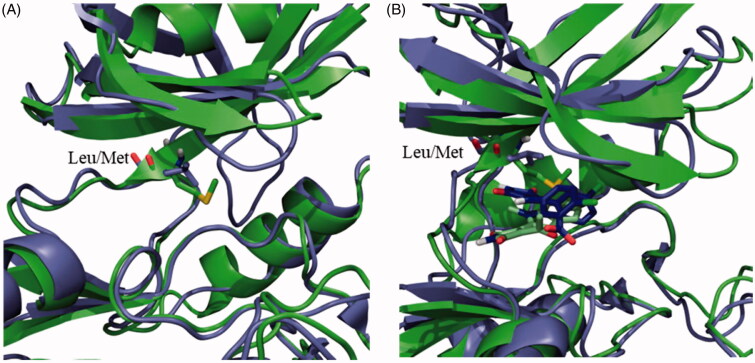
(A) Superposition of the human (purple) and *Leishmania* (green) GSK-3 enzymes. Key mutation in the ATP binding site is depicted as sticks. (B) Superposition of the poses of maleimide **13** in the human (purple) and *Leishmania* (green) GSK-3 enzymes.

Compounds **13** and **14** are maleimide derivatives with a high degree of chemical similarity, but with different binding modes to the human enzyme. Maleimide **13** is a reversible ATP-competitive inhibitor, while maleimide **14** is an irreversible ATP-competitive inhibitor of hGSK-3β due to its halomethylketone tail^45^. Significantly, **13** did not inhibit LdGSK-3 activity, as the compound cannot reach the deep part of the ATP binding cavity, in agreement with docking studies (Figure 2(B)). According to a crystal structure available for the hGSK-3 (PDB code 1Q4L)^64^, the maleimide scaffold interacts with the Asp133 through a hydrogen bond at the deepest part of the ATP binding cavity. This Asp residue is replaced with Glu101 at the LdGSK-3 (Figures S1). This conservative replacement did not significantly modify the structure of the binding site, as it is the backbone that is mostly responsible for the interaction with the inhibitor and not the lateral chain that points outwards the active site of the enzyme, making the number of its methylene groups irrelevant for this interaction. In contrast, its adjacent Leu132 in the human enzyme is mutated by the bulkier Met100 in the *Leishmania* enzyme (Figure 2(A)). This change jeopardises the access of **13** into the deepest part of the cavity, severely precluding the enzyme inhibition. On the other hand, the inhibition by the irreversible inhibitor **14** is lower for LdGSK-3 respect to the human enzyme, despite its *IC*_50_ at low micromolar concentrations. According to the regular free docking results, the methylene group attached to the bromine in **14** is oriented towards the SH of the Cys169 with a distance of 3.7 Å. The orientation of the molecule and the distance suggest an irreversible inhibition of the enzyme. This fact was confirmed in the covalent docking, showing a distance of 1.8 Å between the methylene and the sulphur, typical of this class of bond.

Finally, quinoline **11**, an allosteric inhibitor of hGSK-3β was inactive in LdGSK-3. The cation-π interaction of the quinoline ring with Arg209 is key in the inhibition of the human enzyme^43^. This Arg is replaced with Pro178 in LmjGSK-3 (See sequence alignment in Figures S1). The change of the cationic Arg by the neutral Pro not only modifies the electrostatic environment of this pocket, but also its rigidity; as Pro imposed a strong conformational constraint, absent in the flexible Arg. Thus, this single mutation ruled out the inhibition of LdGSK-3 by **11**.

### Leishbox library as a source of LdGSK-3 inhibitors

In the quest for novel scaffolds of LdGSK-3 inhibitors, the previous search inside putative collections of GSK-3 inhibitors was complemented with an inverse strategy, the search for LdGSK-3 inhibitors inside the 186 compounds belonging to the Leishbox collection^38^. All the kinetoboxes, including Leishbox, were selected from unbiased phenotypic screening. Further studies have been reported recently using kinetoboxes, aimed at expanding the pharmacological potential of these compounds^65–67^. However, studies focussed on the experimental identification of the mechanism of action of these compounds are still scarce^68–70^.

The compounds (**25–210**) were assayed at 10 µM for *in vitro* LdGSK-3 inhibition. Nine molecules (**69**, **71**, **95**, **119**, **124**, **128**, **151**, **184** and **187**) showed a percentage of inhibition higher than 50% being selected for subsequent *IC*_50_ calculation (Table S2). Despite repetitive experiments, only seven resulted with a reliable *IC*_50_ inside the submicromolar or low micromolar ranges (Table 2). In addition, the selectivity of these hits was assessed by hGSK-3β inhibition. The *IC*_50_ values for both enzymes were quite similar most likely due to their binding into the highly preserved ATP binding site of the enzymes. Consequently, the discrimination between human and leishmanial GSK-3 was rather poor (Table 2).

**Table 2.** *IC*_50_ values of hGSK-3 and LdGSK-3 inhibition for hit compounds **71**, **95**, **119**, **124**, **128**, **151** and **187**.

Compound (Leishbox ID)	Chemical structure	LdGSK-3^a^ *IC*_50_ (μM)	hGSK-3β^b^ *IC*_50_ (μM)
**71** (TCMDC-143396)		3.60^c^	1.23 ± 0.14
**95** (TCMDC-143483)		0.46 ± 0.07	1.40 ± 0.25
**119** (TCMDC-143281)		0.27^c^	0.24 ± 0.04
**124** (TCMDC-143391)		6.00^c^	0.81 ± 0.34
**128** (TCMDC-143280)		1.70^c^	1.32 ± 0.35
**151** (TCMDC-143197)		2.54 ± 0.48	0.02 ± 0.01
**187** (TCMDC-143181)		9.10^c^	1.22 ± 0.43

^a^ Indirubin-3’-monoxime-5-sulphonic acid was used as reference of the assay: *IC*_50_ (LdGSK-3) = 2.4 ± 0.2 μM.

^b^ *N*-(4-methoxybenzyl)-*N*′-(5-nitro-1,3-thiazol-2-yl)urea (AR-A01448) was used as reference of the assay: *IC*_50_ (hGSK-3β) = 0.10 ± 0.03 μM.

^c^ Standard deviation not calculated due to experimental issues.

The chemical structures of these 7 compounds belong to three different chemical families: benzoimidazole (**71** and **187**), *N*-phenylpyrimidine-2-amine (**95**, **119**, **124,** and, **128**) and oxadiazole (**151**). Some initial structural insights can be established from the active and inactive members of these three families, compiled in Table S3–S5. For *N*-phenylpyrimidine-2-amines, active compounds lack substitution at 5 and 6 position and showed an aromatic substituent in position 3, together with a free amino group. With regard to benzoimidazole scaffold, only the two derivatives with a 5-phenylpyridin-2-amine (**71** and **187**) are active, highlighting to the importance of this substituent to inhibit GSK-3. Only a single oxadiazole (**151**) showed a significant inhibition against LdGSK-3; this fact, together with the poor representation of this scaffold in the Leishbox, precluded the establishment of a reliable structure-activity relationship. Consequently, we focussed on the other two families. Molecular modelling studies were carried out with *N*-phenylpyrimidine-2-amines and benzoimidazoles to infer their mode of binding and their mechanism of action. In a first step, an unbiased blind docking was performed for one member of each family in order to identify the most likely region of LmjGSK-3 that interacts with the inhibitor. As suspected by their similar *IC*_50_ values for *Leishmania* and human GSK-3, both chemical families interact with the ATP binding site.

**Figure 3.**
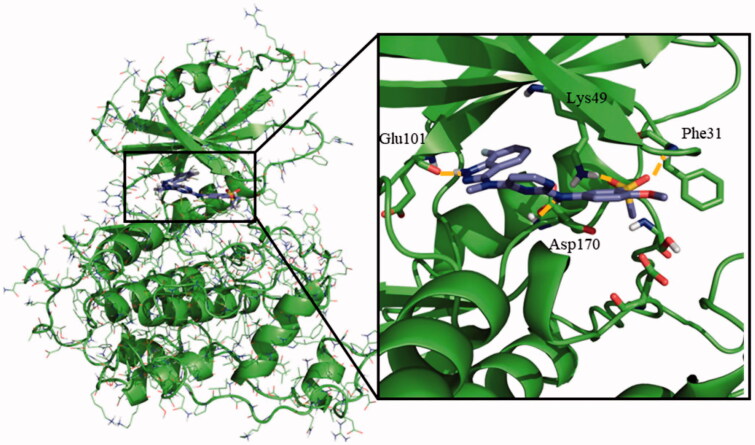
Proposed binding mode for compound **95** with LmjGSK-3 (PDB code 3E3P) with the modelled decapeptide loop. Zoom of ATP binding pocket showed the most relevant interactions of **95** with nearby residues of the binding site.

Once the theoretical binding site was identified, induced fit docking studies were carried out with one of the most active compounds, the pyrimidine **95** (*IC*_50_ = 0.46 µM), in order to optimise the interaction of the residues involved in the binding of this inhibitor. The rationale underlying this step is the readjustment of the protein from its apo form into a holo state. Induced fit docking defined a key hydrogen bond between Glu101 and the amino group from the indazole moiety of compound **95**. Additionally, the sulphone group was able to establish a double hydrogen bond with both Phe31 and Lys49, being the latter a catalytic residue of the protein (Figure 3).

**Figure 4.**
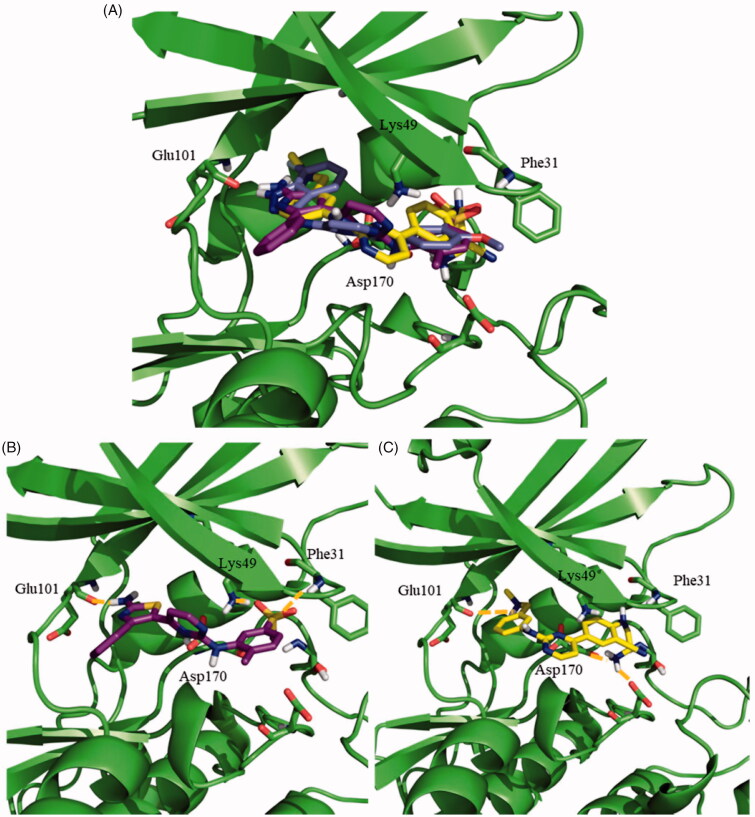
Docking results for the *N*-phenylpyrimidine-2-amines into modelled LmjGSK-3. (A) Superposition of compounds **124** (magenta), **119** (yellow) and **95** (purple) on LmjGSK-3 protein. Key residues were labelled. (B) Binding mode of compound **124**. The main interactions were highlighted. (C) Binding mode of compound **119** showing the main interactions found in the complex.

Afterwards, the docking of the other *N*-phenylpyrimidine-2-amine derivatives **119**, **124** and **128**, was carried out using Autodock. These three compounds maintained the key hydrogen bond of their indazole group with Glu101, as described for **95**. The merging of the most representative docking poses for the different *N*-phenylpyrimidine-2-amines is represented in Figure 4(A). The detailed analysis of the resulting poses disclosed a similar binding mode for **95** and **124,** in agreement with their similar structures. The primary amine of the thiazole ring formed a hydrogen bond with Glu101, while the sulphone group preserves its double interaction with Phe31 and Lys49, as shown in Figure 4(B). For **119**, an ionic interaction at physiological pH was established between the cationic tertiary amine and the anionic carboxylic group of the side chain of Glu101. Furthermore, the primary amine group at the other side of the molecule formed a hydrogen bond with Asp170 (Figure 4(C)). The same interactions for **128** inside the active site of LmjGSK-3 were surmised due to its chemical similarity with **119**.

**Figure 5.**
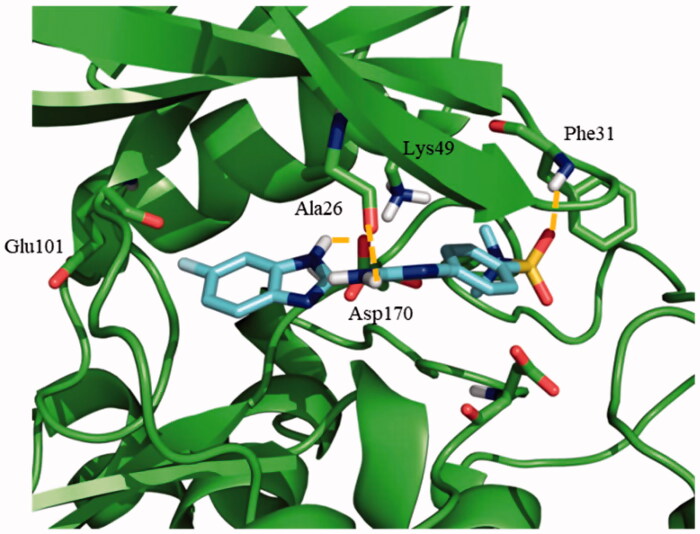
Proposed binding mode for the active benzoimidazole **71** into modelled LmjGSK-3 showing the main interactions found in the complex.

Concerning the binding mode of **71**, as representative of the benzoimidazole family, its sulphone group formed a hydrogen bond with Phe31, while two additional hydrogen bonds were formed between the primary amine with Asp170 and the secondary amine from the benzoimidazole moiety with Ala26, respectively (Figure 5). A similar pattern of interactions was inferred for **187** owing to its chemical similarity with **71.**

As a conclusion, we have broadened the chemical space available for the design of *Leishmania* GSK-3 inhibitors with leishmanicidal properties. With respect to the screening of our in-house library of inhibitors for human PKs, we extended its potential from their former application on human neurodegenerative syndromes into the vast field of infectious diseases. Due to the importance of these pathologies in the developed world, and the huge effort implemented by the big pharma to develop new PKi scaffolds against them, tropical diseases may profit from this trend through a drug repurposing strategy, as has happened with other drugs^71^. With respect to Leishbox, benzoimidazole, *N*-phenylpyrimidine-2-amine, and oxadiazole were uncovered as new scaffolds for LdGSK-3 inhibitors. Moreover, the molecular traits underlying the inhibition of the enzyme by these compounds were disclosed. In this regard, this work succeeded in providing a proof of concept for Leishbox as a source of inhibitors for *Leishmania* GSK-3. Nevertheless, their chemotherapeutical potential will need further validation; for instance, an endorsement of their activities according to the class of macrophages^66^. Secondly, the poor inhibitory selectivity between host and parasite GSK-3 displayed by the molecules must be tackled in future generations of compounds. Even so, hGSK-3β inhibition is tolerated in mammalian cells evidence by the clinical trials under current progress for human pathologies^60^. Moreover, the inhibition of hGSK-3 led to an anti-inflammatory effect, which may be beneficial for the excessive pathological inflammatory response frequently associated with this disease^72^^,^^73^. A feasible solution to increase the specificity of action for these inhibitors would be to vehicle them through the endocytic pathway, hence into the parasitophorous vacuole. This would promote the preferential accumulation of the inhibitor in the vicinity of the parasite, an approach widely used for this pathology, including the liposomal formulation of amphotericin B, preventing the strong toxicity of the free drug. Work pursuing these goals is currently in progress.

## Supplementary Material

Supplemental Material
