# Supplementary material for: Towards discovery of new leishmanicidal scaffolds able to inhibit Leishmania GSK-3
Source: J Enzyme Inhib Med Chem. 2019 Nov 22;35(1):199–210. doi: 10.1080/14756366.2019.1693704 (PMC6882465; doi:10.1080/14756366.2019.1693704)
Supplement: Supplemental Material [file IENZ_A_1693704_SM6011.pdf]

Supporting Information for:

Towards discovery of new leishmanicidal scaffolds able to inhibit *Leishmania* GSK-3

Paula Martínez de Iturrate,[a] Victor Sebastián-Pérez,[a] Montserrat Nácher-Vázquez,[a] Catherine S. Tremper,[a] Despina Smirlis,[b] Julio Martín,[c] Ana Martínez,[a] Nuria E. Campillo,[a] Luis Rivas[a,*] and Carmen Gil[a,*]

[a]Centro de Investigaciones Biológicas (CIB-CSIC), Madrid, Spain

[b]Hellenic Pasteur Institute, Athens, Greece

[c]Global Health R&D, GlaxoSmithKline, Tres Cantos, Spain

Contents:

- Page S2: Figure S1
- Page S3: Figure S2
- Page S4: Table S1
- Page S9: Table S2
- Page S32: Table S3
- Page S35: Table S4
- Page S37: Table S5
- Page S38: References

```
sp|P49841|GSK3B_HUMAN   MSGRPRTTSFAESCKPVQQPSAFGSMKVSRDKDGSKVTTVVATPGQGPDRPQEVSYTDTK 60
tr|Q4QE15|Q4QE15_LEIMA  ------------------------------------MSLNAAAAADERSRKEMDRFQVER 24
                                                            ::  .*  .:  .* :   :   :

sp|P49841|GSK3B_HUMAN   VIGNGSFGVVYQAKLCDSGELVAIKKVLQDKRFKNRELQIMRKL---DHCNIVRLRYFFY 117
tr|Q4QE15|Q4QE15_LEIMA  MAGQGTFGTVQLGKEKSTGMSVAIKKVIQDPRFRNRELQIMQDLAVLHHPNIVQLQSYFY 84
                        : *:*:**.*  .*  .:*  ******:** **:*******:.*   .* ***:*: :**

sp|P49841|GSK3B_HUMAN   SSGEK-KDEVYLNLVLDYVPETVYRVARHYSRAKQTLPVIYVKLYMYQLFRSLAYIH--S 174
tr|Q4QE15|Q4QE15_LEIMA  TLGERDRRDIYLNVVMEYVPDTLHRCCRNYYRRQVAPPPILIKVFLFQLIRSIGCLHLPS 144
                        : **: : ::***:*::***:*::* .*:* * : : * * :*::::**:**:. :*  *

sp|P49841|GSK3B_HUMAN   FGICHRDIKPQNLLLDPDTAVLKLCDFGSAKQLVRGEPNVSYICSRYYRAPELIFGATDY 234
tr|Q4QE15|Q4QE15_LEIMA  VNVCHRDIKPHNVLVNEADGTLKLCDFGSAKKLSPSEPNVAYICSRYYRAPELIFGNQHY 204
                        ..:*******:*:*::   ..**********:*  .****:***************  .*

sp|P49841|GSK3B_HUMAN   TSSIDVWSAGCVLAELLLGQPIFPGDSGVDQLVEIIKVLGTPTREQIREMNPNYTEFKFP 294
tr|Q4QE15|Q4QE15_LEIMA  TTAVDIWSVGCIFAEMMLGEPIFRGDNSAGQLHEIVRVLGCPSREVLRKLNPSHTDVDLY 264
                        *:::*:**.**::**::**:*** **....** **::*** *:** :*::**.:*:..:

sp|P49841|GSK3B_HUMAN   QIKAHPWTKVFRPRT---PPEAIALCSRLLEYTPTARLTPLEACAHSFFDELRDPNVKLP 351
tr|Q4QE15|Q4QE15_LEIMA  NSKGIPWSNVFSDHSLKDAKEAYDLLSALLQYLPEERMKPYEALCHPYFDELHDPATKLP 324
                        : *. **:.**  ::     **  * * **:* *  *:.* ** .* :****:*  .***

sp|P49841|GSK3B_HUMAN   NGRDTPA-LFNFTTQELSSNPPLATILIPPHARIQAAASTPTNATAASDANTGDRGQTNN 410
tr|Q4QE15|Q4QE15_LEIMA  NNKDLPEDLFRFLPNEIEVMSEAQKAKLVRK----------------------------- 355
                        *.:* *  **.*  .*:.      .  :  :

sp|P49841|GSK3B_HUMAN   AASASASNST 420
tr|Q4QE15|Q4QE15_LEIMA  ---------- 355
```

Figure S1. Sequence alignment of the GSK-3 structures of *Leishmania major* (Q4QE15) and *Homo sapiens* (P49841).

```
tr|A0A088RMG0_9TRYP  MSLNAADAADDRSRKEMDRFQVERMAGQGTFGTVQLGKEKSTGMSVAIKKVIQDPRFRNR   60
tr|Q4QE15_LEIMA      MSLNAAAAADERSRKEMDRFQVERMAGQGTFGTVQLGKEKSTGMSVAIKKVIQDPRFRNR   60
tr|A6N857_LEIDO      MSLNAADAADERSRKEMDRFQVERMAGQGTFGTVQLGKEKSTGMSVAIKKVIQDPRFRNR   60
tr|A4HXQ3_LEIIN      MSLNAADAADERSRKEMDRFQVERMAGQGTFGTVQLGKEKSTGMSVAIKKVIQDPRFRNR   60
tr|Q0PKV3_LEIME      MSLNAADAADERSRKEMDRFLVERMAGQGTFGTVQLGKEKSTGMSVAIKKVIQDPRFRNR   60
                     ****** ***:********* ***************************************

tr|A0A088RMG0_9TRYP  ELQIMQDLAVLHHPNIVQLQSYFYTLGERDRRDIYLNVVMEYVPDTLHRCCRNYYRRQTT   120
tr|Q4QE15_LEIMA      ELQIMQDLAVLHHPNIVQLQSYFYTLGERDRRDIYLNVVMEYVPDTLHRCCRNYYRRQVA   120
tr|A6N857_LEIDO      ELQIMQDLAVLHHPNIVQLQSYFYTLGERDRRDIYLNVVMEYVPDTLHRCCRNYYRRQVA   120
tr|A4HXQ3_LEIIN      ELQIMQDLAVLHHPNIVQLQSYFYTLGERDRRDIYLNVVMEYVPDTLHRCCRNYYRRQVA   120
tr|Q0PKV3_LEIME      ELQIMQDLAVLHHPNIVQLQSYFYTLGERDRRDIYLNVVMEYVPDTLHRCCRNYYRRQVA   120
                     **********************************************************.:

tr|A0A088RMG0_9TRYP  PPPILIKVFLFQLIRSIGCLHLPSVNVCHRDIKPHNVLVNEAEGTLKLCDFGSAKKLSPS   180
tr|Q4QE15_LEIMA      PPPILIKVFLFQLIRSIGCLHLPSVNVCHRDIKPHNVLVNEADGTLKLCDFGSAKKLSPS   180
tr|A6N857_LEIDO      PPPILIKVFLFQLIRSIGCLHLPSVNVCHRDIKPHNVLVNEADGTLKLCDFGSAKKLSPS   180
tr|A4HXQ3_LEIIN      PPPILIKVFLFQLIRSIGCLHLPSVNVCHRDIKPHNVLVNEADGTLKLCDFGSAKKLSPS   180
tr|Q0PKV3_LEIME      PPPILIKVFLFQLIRSIGCLHLPSVNVCHRDIKPHNVLVNEAEGTLKLCDFGSAKKLSPS   180
                     ******************************************:*****************

tr|A0A088RMG0_9TRYP  EPNVAYICSRYYRAPELIFGNQHYTTAVDIWSVGCIFAEMMLGEPIFRGDNSAGQLHEIV   240
tr|Q4QE15_LEIMA      EPNVAYICSRYYRAPELIFGNQHYTTAVDIWSVGCIFAEMMLGEPIFRGDNSAGQLHEIV   240
tr|A6N857_LEIDO      EPNVAYICSRYYRAPELIFGNQHYTTSVDIWSVGCIFAEMMLGEPIFRGDNSAGQLHEIV   240
tr|A4HXQ3_LEIIN      EPNVAYICSRYYRAPELIFGNQHYTTSVDIWSVGCIFAEMMLGEPIFRGDNSAGQLHEIV   240
tr|Q0PKV3_LEIME      EPNVAYICSRYYRAPELIFGNQHYTTSVDIWSVGCIFAEMMLGEPIFRGDNSAGQLHEIV   240
                     **************************:*********************************

tr|A0A088RMG0_9TRYP  RVLGCPPREVLRKLNPSHTDVDLYNSKGIPWSNVFCDQSLKDAKEAHDLLSGLLQYLPEE   300
tr|Q4QE15_LEIMA      RVLGCPSREVLRKLNPSHTDVDLYNSKGIPWSNVFSDHSLKDAKEAYDLLSALLQYLPEE   300
tr|A6N857_LEIDO      RVLGCPSREVLRKLNPSHTDVDLYNSKGIPWSSVFCDHSLKDAKEAYDLLSALLQYLPED   300
tr|A4HXQ3_LEIIN      RVLGCPSREVLRKLNPSHTDVDLYNSKGIPWSSVFCDHSLKDAKEAYDLLSALLQYLPED   300
tr|Q0PKV3_LEIME      RVLGCPSREVLRKLNPSHTDVDLYNSKGIPWSTVFCDHSLKDAKEAYDLLSALLQYLPED   300
                     ****** *************************.**.*:********:****.*******:

tr|A0A088RMG0_9TRYP  RTKPYEALCHPYFDELRDSATKLPNNKDLPDDLFHFLPAEVEIMTDVQKGKLIRK   355
tr|Q4QE15_LEIMA      RMKPYEALCHPYFDELHDPATKLPNNKDLPEDLFRFLPNEIEVMSEAQKAKLVRK   355
tr|A6N857_LEIDO      RMKPYEALCHPYFDELHDSATKLPNNKDLPEDLFRFLPSEIEVMSEAQKAKLVRK   355
tr|A4HXQ3_LEIIN      RMKPYEALCHPYFDELHDSATKLPNNKDLPEDLFRFLPSEIEVMSEAQKAKLVRK   355
tr|Q0PKV3_LEIME      RMKPYEALCHPYFDELHDSATKLPNHKNLPEDLFRFLPSEIEVMSEAQKAKLVRK   355
                     * **************:* ******:*:**:***:*** *:*:*::.**.**:**
```

```
#  Percent Identity  Matrix
#
#

     1: tr|A0A088RMG0|A0A088RMG0_9TRYP  100.00   93.52   93.52   93.52   92.96
     2: tr|Q4QE15|Q4QE15_LEIMA           93.52  100.00   98.03   98.03   96.90
     3: tr|A6N857|A6N857_LEIDO           93.52   98.03  100.00  100.00   98.59
     4: tr|A4HXQ3|A4HXQ3_LEIIN           93.52   98.03  100.00  100.00   98.59
     5: tr|Q0PKV3|Q0PKV3_LEIME           92.96   96.90   98.59   98.59  100.00
```

Figure S2. Sequence alignment of *Leishmania spp.* GSK-3 sequences available in the Uniprot database. The identity index among the sequences is also included. *L. panamensis* (A0A088RMG0)*, L. major* (Q4QE15)*, L. donovani* (A6N857)*, L. infantum* (A4HXQ3) and *L. mexicana* (Q0PKV3).

Table S1. *In vitro* enzymatic and antiparasitic activities of first selection of hGSK-3β inhibitors (**1-24**).[a]

Compound	Chemical structure	hGSK-3β IC_{50} (μM)	LdGSK-3[b] IC_{50} (μM)	*L. infantum* promastigotes EC_{50} (μM)	*L. pifanoi* amast. ax EC_{50} (μM)	PMM[c] EC_{50} (μM)	SI[d]
**1**	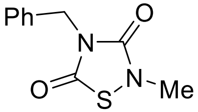	2[1]	1.1±0.2	10.9±0.4	2.0±1.9	32.9±3.5	16.5
**2**	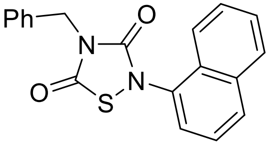	0.005[2]	0.32±0.05	17.6±2.3	7.1±1.8	>50	>7.0
**3**	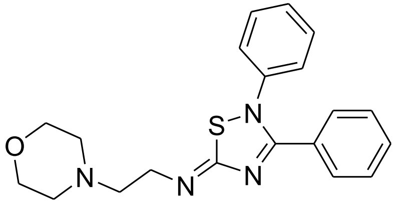 2HBr	0.9±0.1[3]	0.24±0.00	>25	>50	-	-
**4**	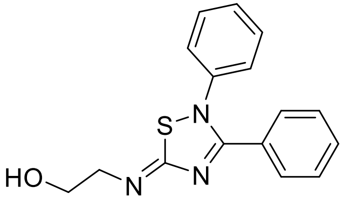 HBr	2.0±0.4[3]	0.17±0.00	>25	>50	-	-
**5**	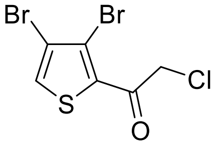	0.5[4]	1.8±0.3	4.6±0.2	2.2±0.6	6.3±1.2	2.9

**6**	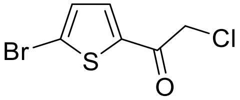	10[4]	12.5±2.4	0.9±0.1	1.7±0.1	6.8±0.4	4.0
**7**	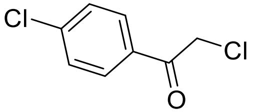	2.5[4]	7.5±1.4	6.6±0.5	0.5±0.1	7.3±1.0	14.6
**8**	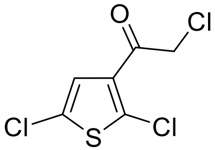	5[4]	10.3±2.0	4.4±0.1	2.4±0.4	3.6±0.5	1.5
**9**	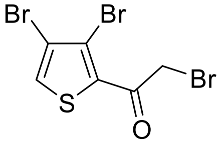	1.0[5]	74.3%@10 μM	>50	>50	-	-
**10**	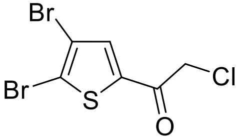	1[4]	3.0±0.4	8.6±0.3	1.2±0.2	7.9±0.6	6.6
**11**	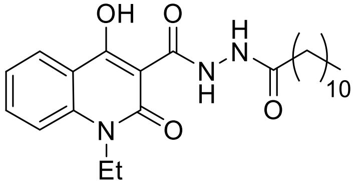	3.01±0.14[6]	<20%@10 μM	>50	3.6±1.3	9.9±0.9	2.8
**12**	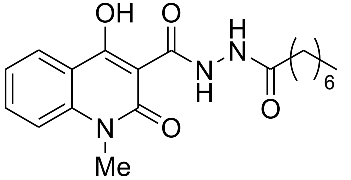	8.7±0.4[7]	<20%@10 μM	>50	20.8±0.0	-	-

**13**	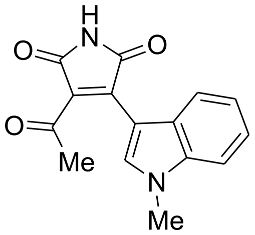	0.89±0.19[8]	<20%@10 μM	>50	>25	-	-
**14**	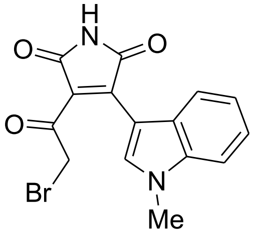	0.005±0.001[8]	1.6±0.2 μM	>50	6.5±2.0	>25	>3.8
**15**	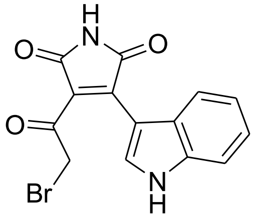	0.047±0.007[8]	17.7±2.7	>50	>50	-	-
**16**	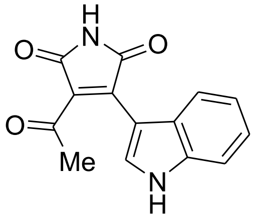	4.47±0.35[8]	<20%@10 μM	>50	2.5±2.7	>50	>20.0

**17**	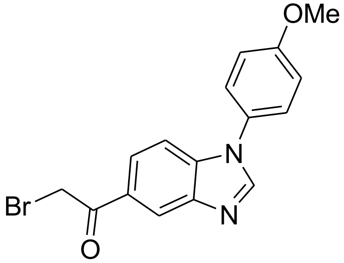	0.58±0.07[8]	35.5%@10 µM	3.4±0.5	4.5±0.2	>25	>5.6
**18**	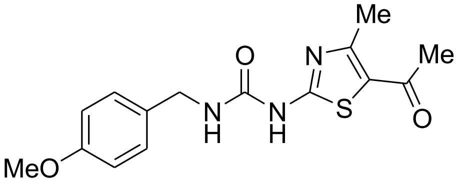	0.8[9]	<20%@10 µM	>50	>50	-	-
**19**	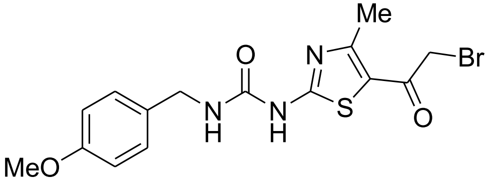	9.72±0.57	<20%@10 µM	>25	14.4±2.6	32.0±3.1	2.2
**20**	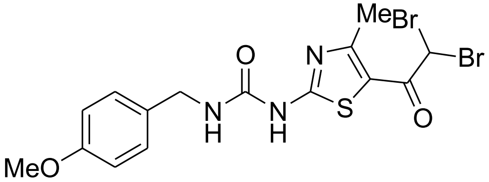	6.22±0.20	<20%@10 µM	>50	>50	-	-
**21**	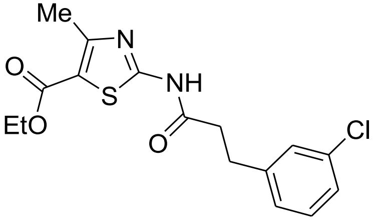	7.23±0.26	<20%@10 µM	>50	>50	-	-

**22**	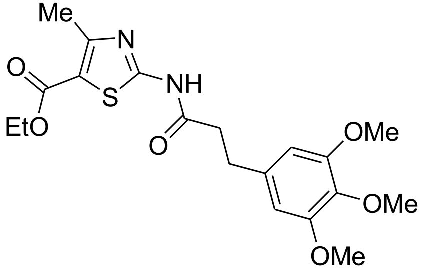	7.04±0.27	<20%@10 µM	>50	>50	-	-
**23**	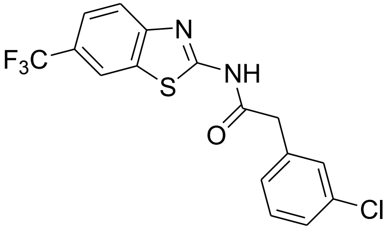	9.75±0.26	<20%@10 µM	>50	>50	-	-
**24**	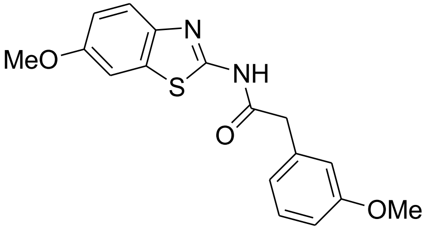	7.33±0.15	<20%@10 µM	>50	>50	-	-

[a]IC_{50}: 50% inhibitory concentration; EC_{50}: 50% effective concentration.
[b]Indirubin-3'-monoxime-5-sulphonic acid was used as reference of the assay: IC_{50} (LdGSK-3)= 2.4±0.2 µM.
[c]PMM: peritoneal murine macrophages.
[d]SI: Specificity Index (EC_{50} PMM/EC_{50} amas. ax).

Table S2. Chemical structure of the Leishbox compounds (**25-210**) and the initial screening on LdGSK3.

Compd.	Leishbox ID	Chemical structure	% inhibition @ 10μM
**25**	TCMDC-143347	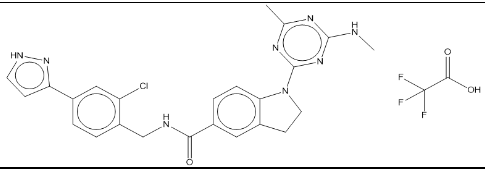	-13.55±3.90
**26**	TCMDC-134026	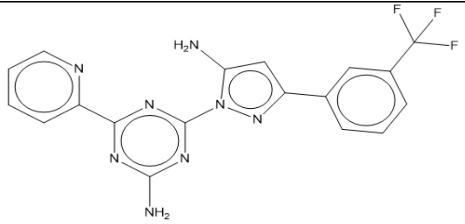	4.75±1.20
**27**	TCMDC-143077	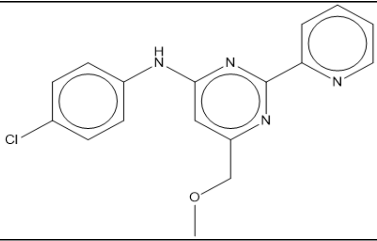	6.35±5.75
**28**	TCMDC-143512	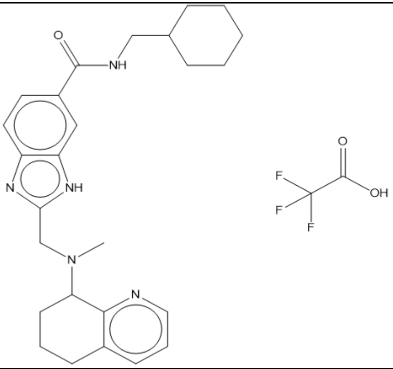	1.15±1.75
**29**	TCMDC-143217	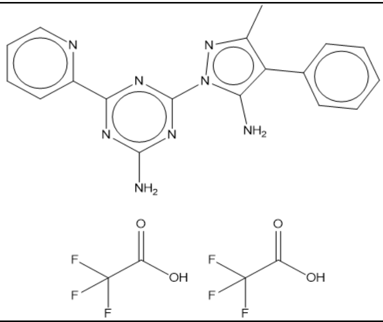	3.80±1.90
**30**	TCMDC-143442	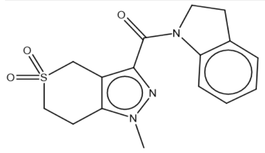	5.20±1.90

**31**	TCMDC-143180	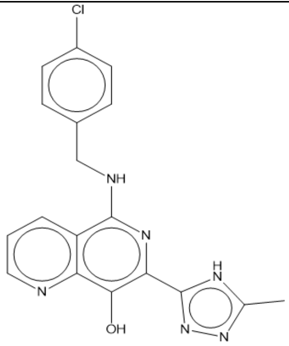	8.20±1.70
**32**	TCMDC-143621	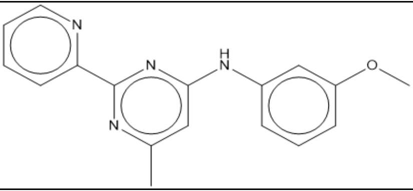	8.80±4.10
**33**	TCMDC-143600	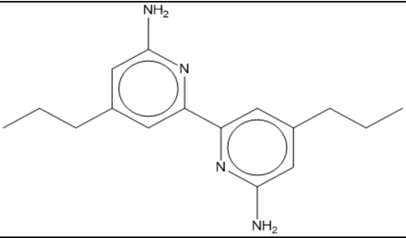	-0.35±3.85
**34**	TCMDC-143211	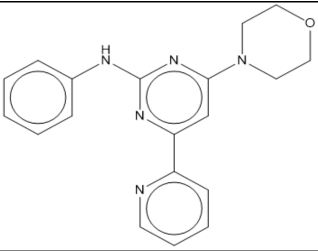	-2.00±2.70
**35**	TCMDC-143078	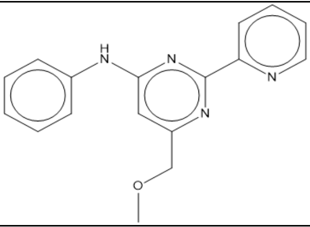	2.90±4.05
**36**	TCMDC-143136	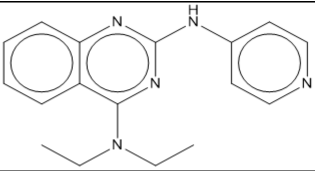	7.10±1.90
**37**	TCMDC-143459	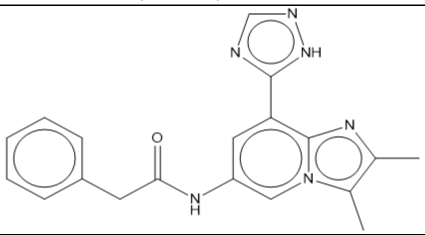	5.90±4.55

**38**	TCMDC-143212	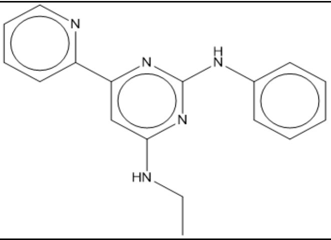	7.10±1.40
**39**	TCMDC-143076	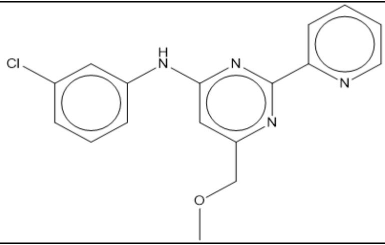	9.00±3.05
**40**	TCMDC-143554	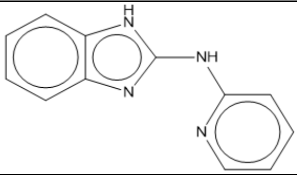	13.50±3.10
**41**	TCMDC-143443	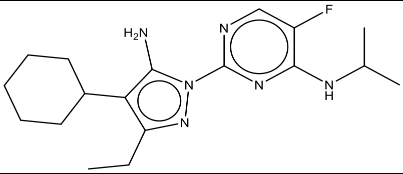	7.05±1.35
**42**	TCMDC-143216	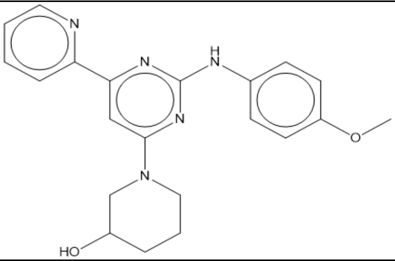	5.30±2.55
**43**	TCMDC-143486	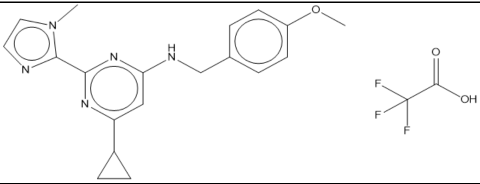	15.30±1.60
**44**	TCMDC-143427	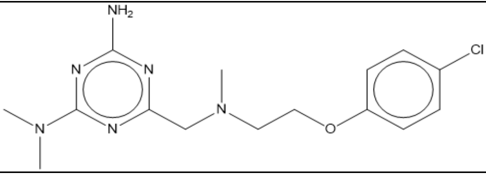	-2.45±1.00
**45**	TCMDC-143213	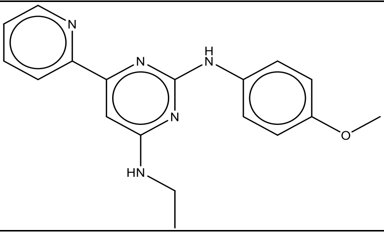	-2.50±5.50

**46**	TCMDC-143260	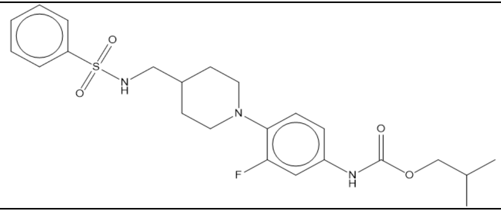	4.95±4.45
**47**	TCMDC-143122	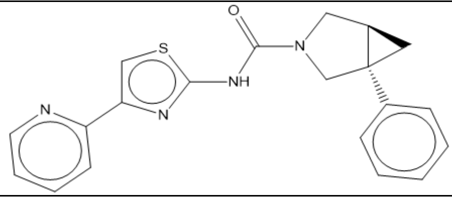	1.55±0.55
**48**	TCMDC-143215	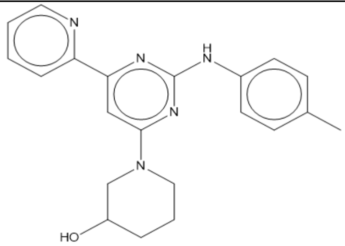	3.25±2.75
**49**	TCMDC-143487	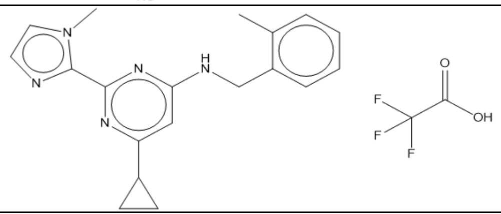	5.60±2.35
**50**	TCMDC-125826	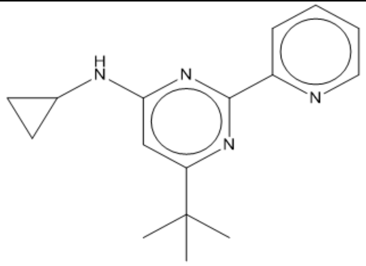	4.70±2.30
**51**	TCMDC-143196	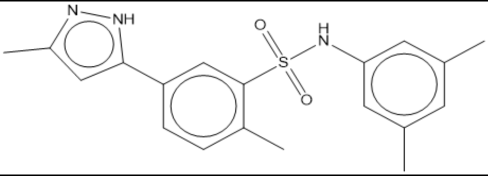	5.95±1.25
**52**	TCMDC-143630	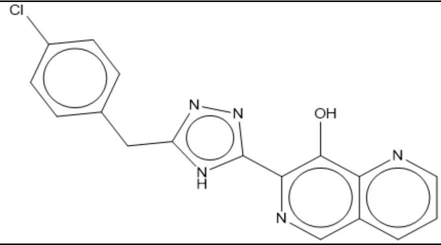	5.05±2.35
**53**	TCMDC-143350	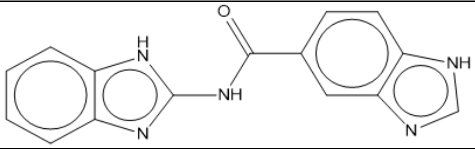	-1.60±1.50
**54**	TCMDC-143113	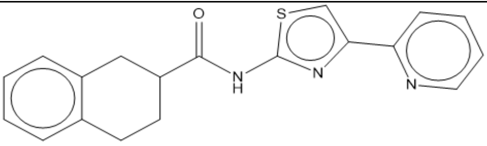	0.10±1.10

**55**	TCMDC-143397	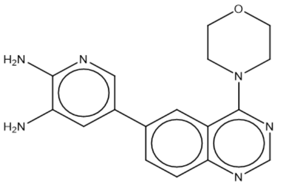	0.55±3.00
**56**	TCMDC-143295	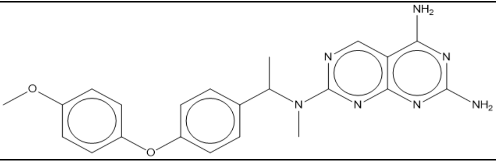	0.50±2.95
**57**	TCMDC-143607	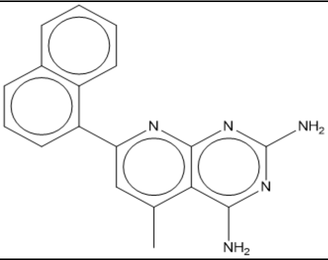	6.10±3.15
**58**	TCMDC-143091	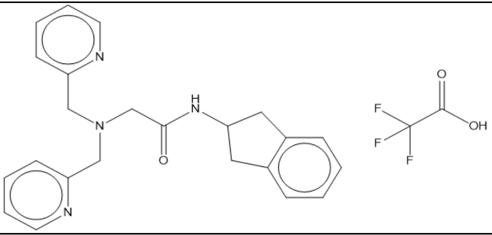	3.75±2.40
**59**	TCMDC-143169	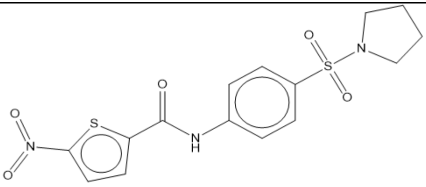	16.15±4.10
**60**	TCMDC-143245	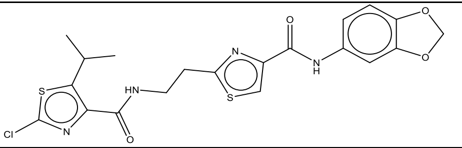	0.20±2.80
**61**	TCMDC-143404	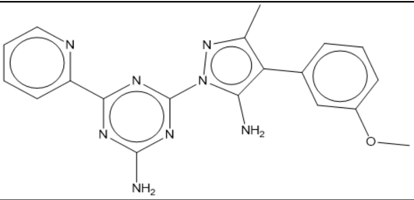	-2.20±1.15
**62**	TCMDC-143296	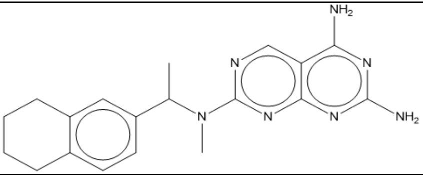	12.10±2.40
**63**	TCMDC-143388	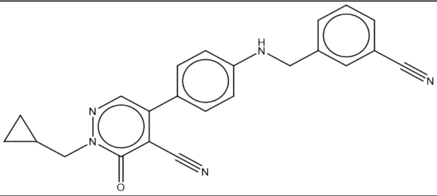	-3.20±0.80
**64**	TCMDC-143133	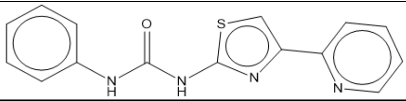	1.40±0.95

**65**	TCMDC-143501	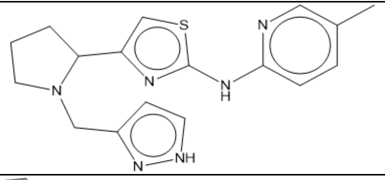	-0.35±2.35
**66**	TCMDC-143633	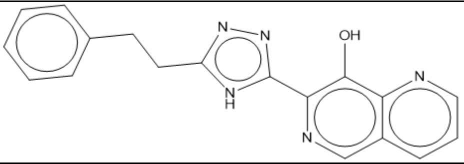	0.90±0.90
**67**	TCMDC-143349	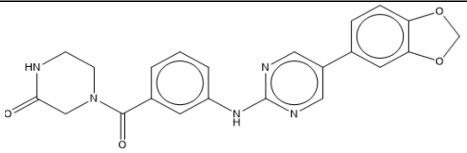	5.65±3.60
**68**	TCMDC-143094	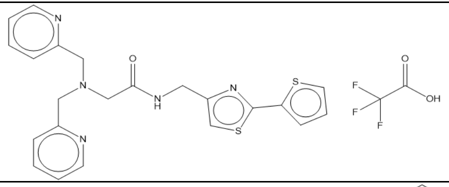	6.80±1.95
**69**	TCMDC-143171	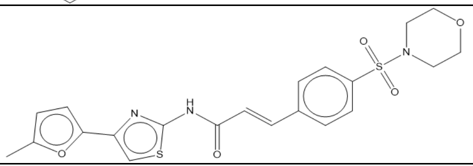	64.27±2.13
**70**	TCMDC-143277	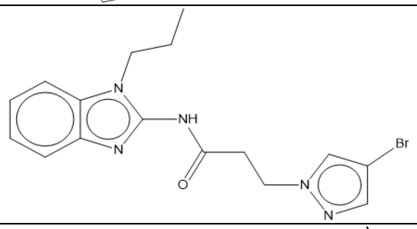	3.80±1.25
**71**	TCMDC-143396	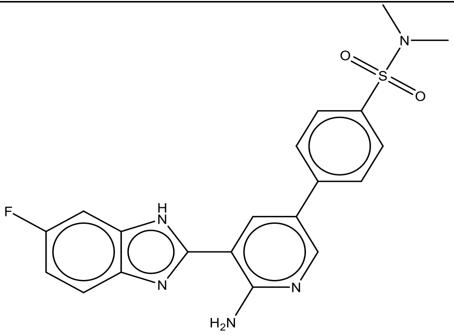	74.63±1.76
**72**	TCMDC-143238	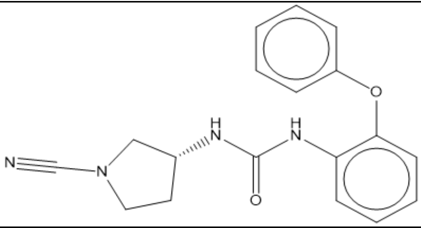	7.40±4.30
**73**	TCMDC-143246	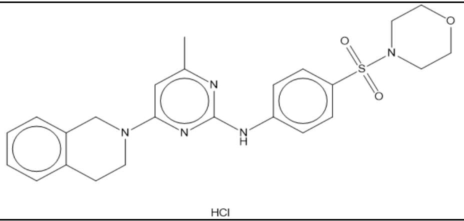	-3.05±0.35

**74**	TCMDC-143451	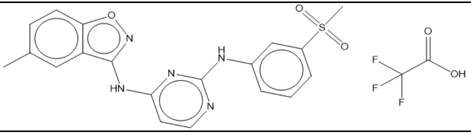	10.00±0.75
**75**	TCMDC-143297	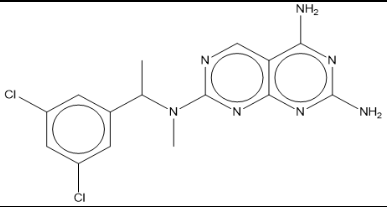	10.05±1.10
**76**	TCMDC-143491	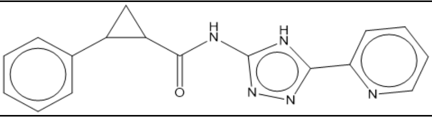	5.70±0.50
**77**	TCMDC-143629	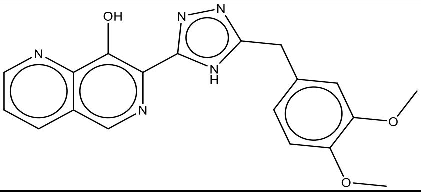	4.60±0.95
**78**	TCMDC-143144	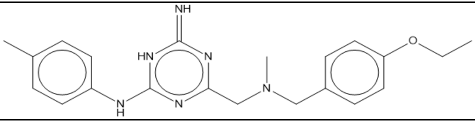	4.90±1.45
**79**	TCMDC-143168	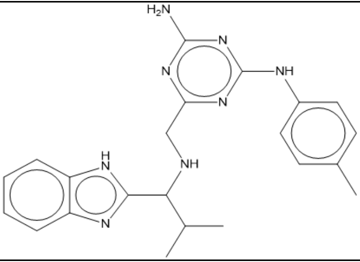	6.40±1.30
**80**	TCMDC-143261	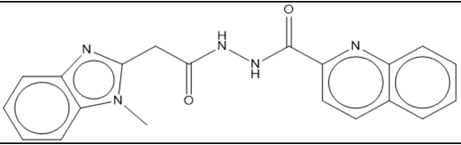	7.95±1.65
**81**	TCMDC-143418	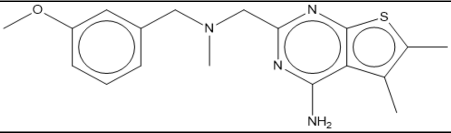	6.35±3.00
**82**	TCMDC-143099	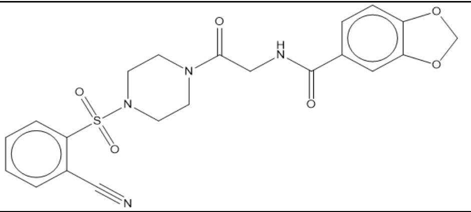	1.90±2.60
**83**	TCMDC-143351	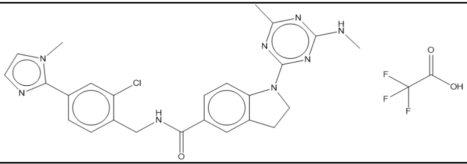	-5.05±0.45

**84**	TCMDC-143285	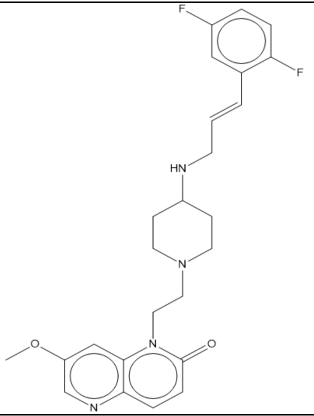	3.55±2.20
**85**	TCMDC-143503	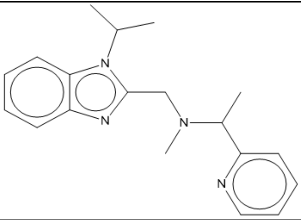	3.45±4.90
**86**	TCMDC-143072	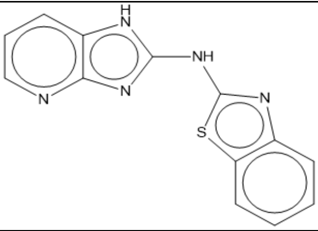	6.55±2.70
**87**	TCMDC-143145	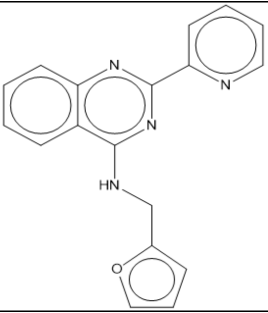	-0.05±0.95
**88**	TCMDC-143115	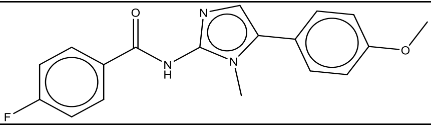	2.00±1.95
**89**	TCMDC-143175	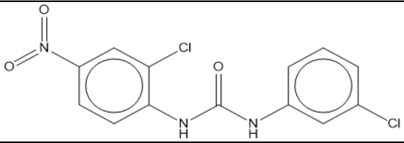	4.80±2.05
**90**	TCMDC-143305	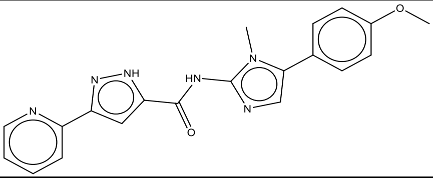	6.40±2.45

**91**	TCMDC-143524	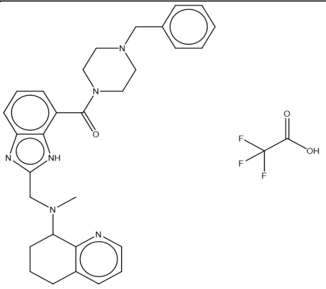	12.65±6.10
**92**	TCMDC-143509	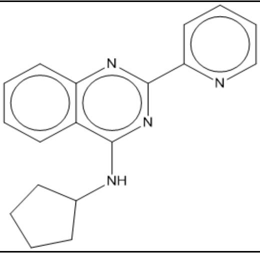	-0.55±1.25
**93**	TCMDC-143375	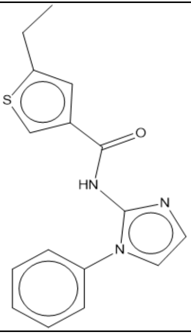	-2.35±1.95
**94**	TCMDC-143406	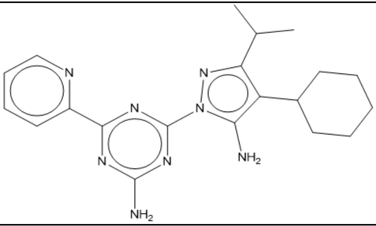	10.45±0.55
**95**	TCMDC-143483	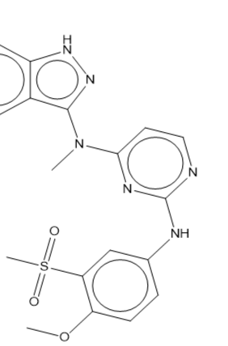	90.25±1.12
**96**	TCMDC-143431	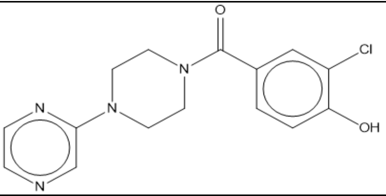	1.65±2.40
**97**	TCMDC-143577	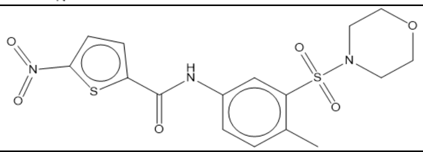	6.15±1.05

**98**	TCMDC-143124	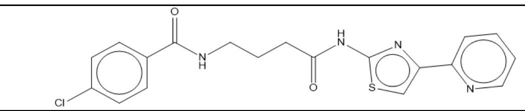	8.50±1.10
**99**	TCMDC-143214	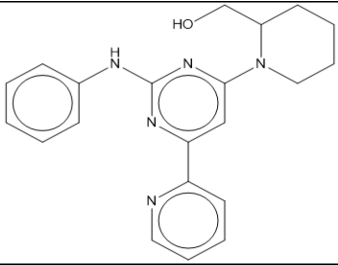	0.60±0.60
**100**	TCMDC-143249	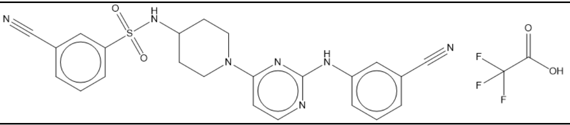	37.05±1.50
**101**	TCMDC-143628	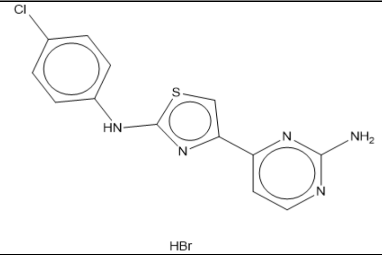	16.40±2.40
**102**	TCMDC-143627	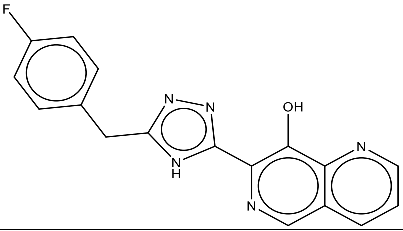	4.05±3.10
**103**	TCMDC-143086	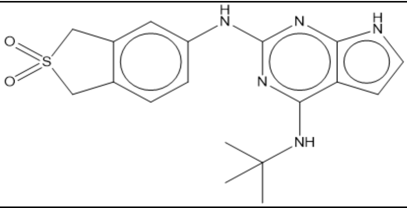	25.85±1.25
**104**	TCMDC-143407	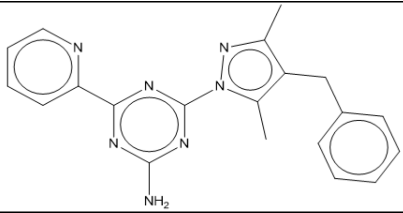	-0.75±1.55
**105**	TCMDC-143398	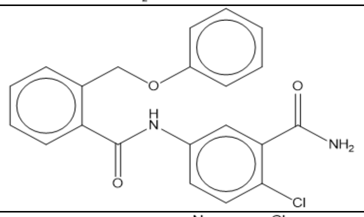	-0.45±1.10
**106**	TCMDC-143419	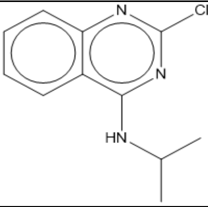	-4.60±0.90

**107**	TCMDC-142900	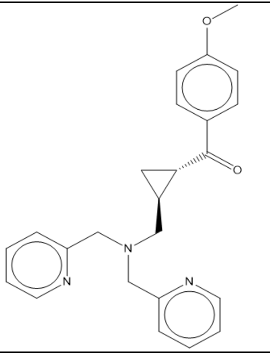	-4.05±3.20
**108**	TCMDC-143141	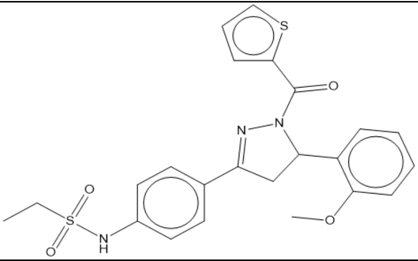	5.15±1.90
**109**	TCMDC-125160	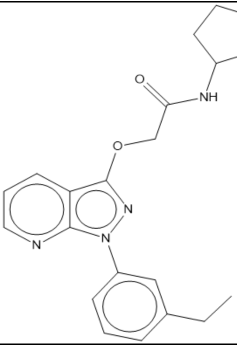	-4.55±1.90
**110**	TCMDC-143274	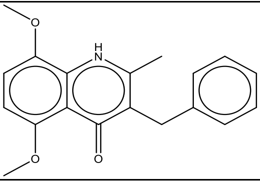	-0.10±5.35
**111**	TCMDC-143448	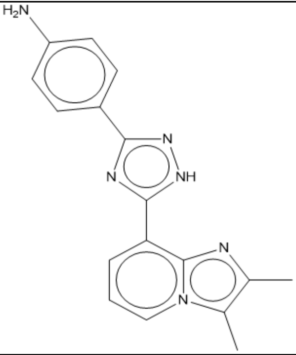	7.35±4.65
**112**	TCMDC-143603	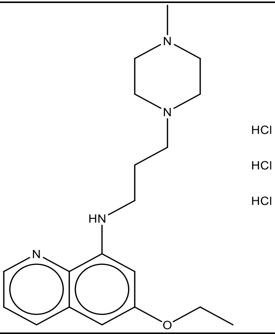	7.50±0.65

**113**	TCMDC-143447	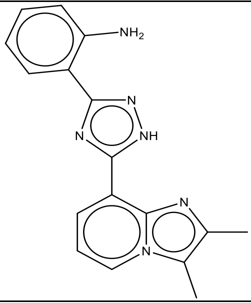	1.40±0.50
**114**	TCMDC-143478	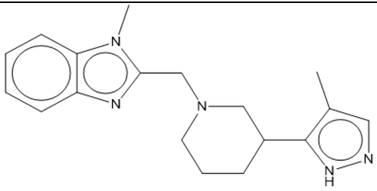	2.85±0.45
**115**	TCMDC-143473	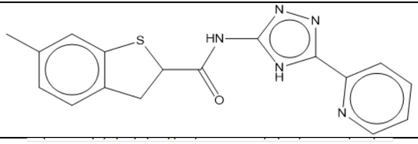	3.20±0.80
**116**	TCMDC-143584	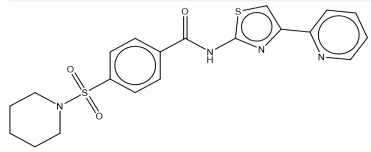	7.05±1.35
**117**	TCMDC-143571	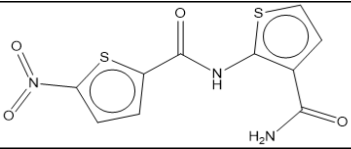	6.20±1.15
**118**	TCMDC-143239	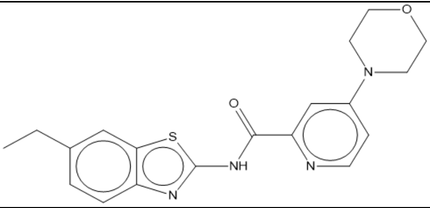	22.80±3.55
**119**	TCMDC-143281	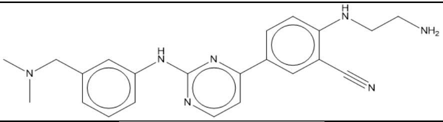	100.29±1.24
**120**	TCMDC-143429	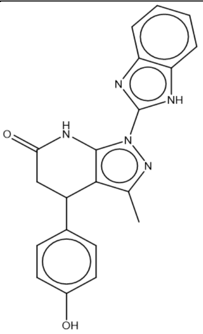	18.15±1.70
**121**	TCMDC-143647	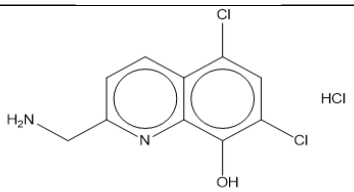	14.80±1.70

**122**	TCMDC-143480	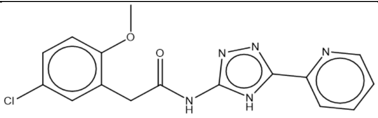	3.95±2.45
**123**	TCMDC-143482	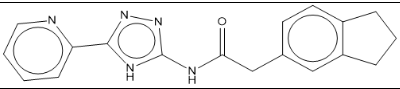	12.15±3.90
**124**	TCMDC-143391	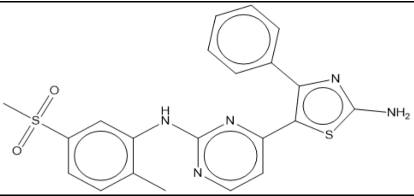	70.59±3.12
**125**	TCMDC-143594	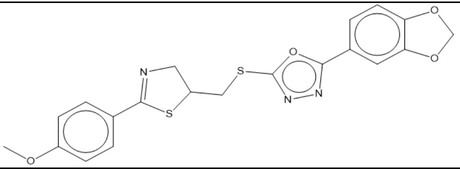	10.60±1.00
**126**	TCMDC-143576	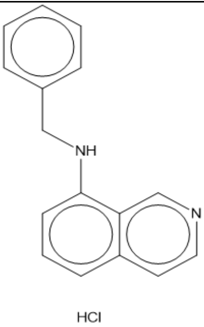	17.10±1.10
**127**	TCMDC-143208	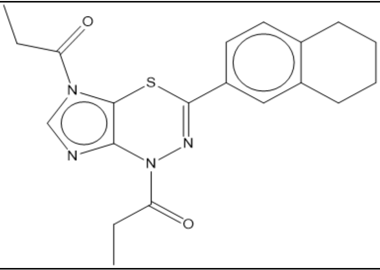	9.65±3.70
**128**	TCMDC-143280	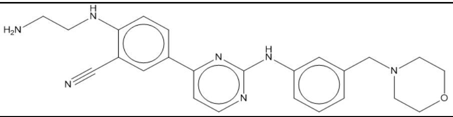	99.46±0.78
**129**	TCMDC-143488	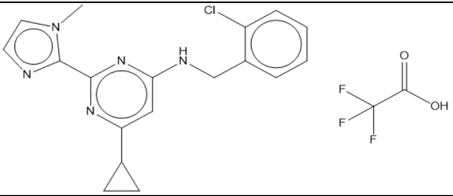	8.90±6.75
**130**	TCMDC-143075	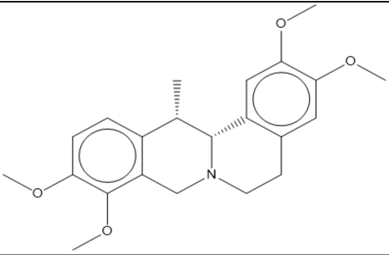	12.85±2.80
**131**	TCMDC-143163	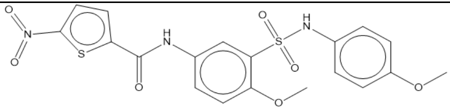	19.75±3.70

**132**	TCMDC-143521	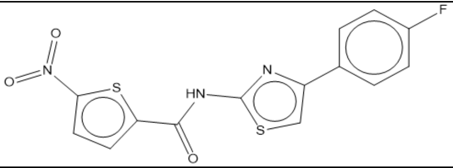	8.15±0.95
**133**	TCMDC-143563	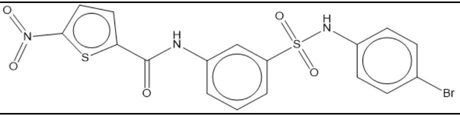	15.75±4.25
**134**	TCMDC-143092	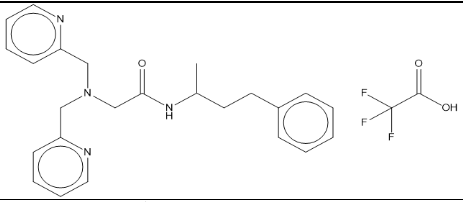	5.25±1.70
**135**	TCMDC-143106	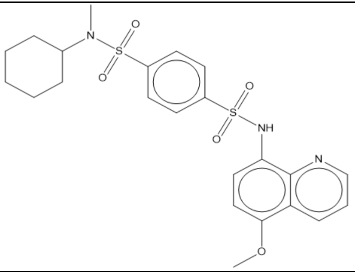	6.85±1.55
**136**	TCMDC-143129	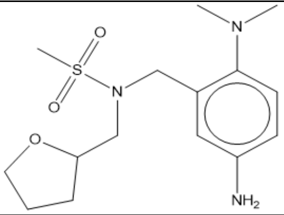	13.95±3.25
**137**	TCMDC-124508	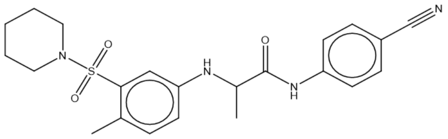	9.35±1.30
**138**	TCMDC-143278	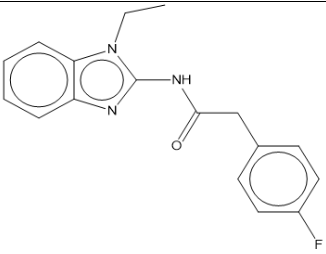	13.00±3.55
**139**	TCMDC-143269	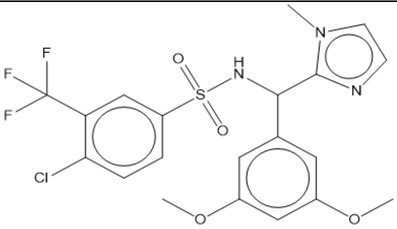	15.95±1.70
**140**	TCMDC-143367	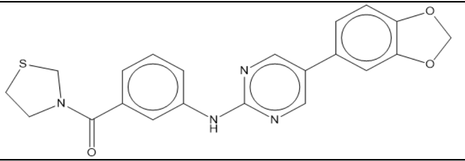	18.20±7.00

**141**	TCMDC-143164	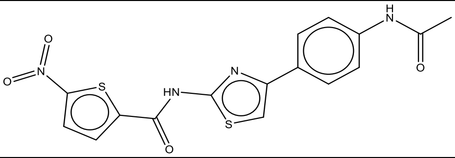	8.55±0.55
**142**	TCMDC-143534	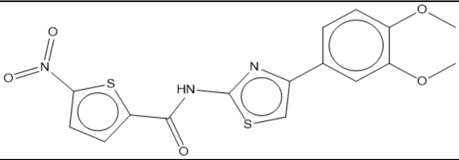	7.95±2.05
**143**	TCMDC-143567	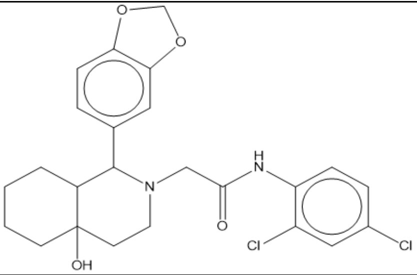	2.25±0.35
**144**	TCMDC-143095	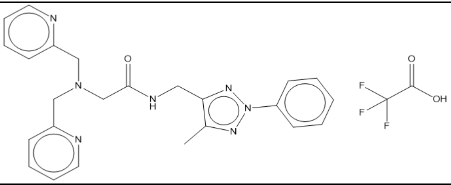	9.10±1.75
**145**	TCMDC-143110	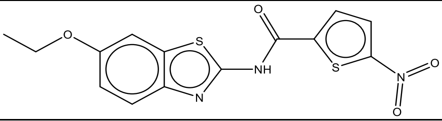	9.25±0.40
**146**	TCMDC-143147	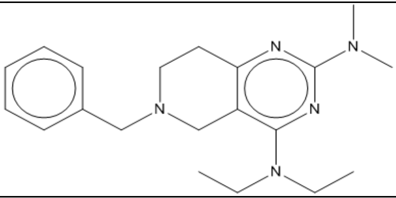	11.85±0.55
**147**	TCMDC-143558	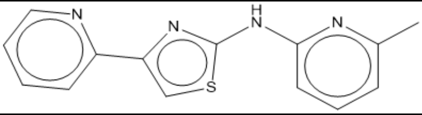	13.40±2.35
**148**	TCMDC-143223	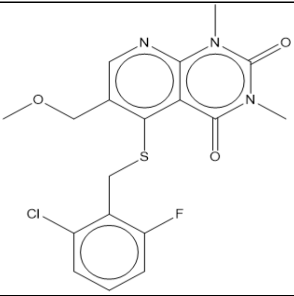	9.60±3.10
**149**	TCMDC-143266	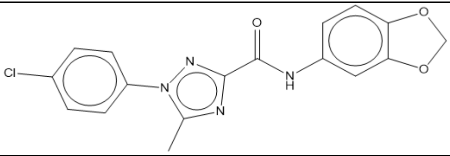	8.90±2.10

**150**	TCMDC-143353	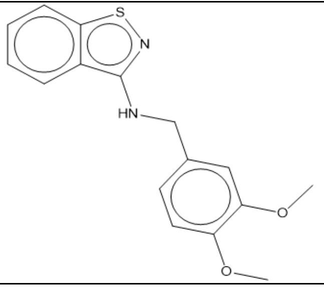	13.55±4.65
**151**	TCMDC-143197	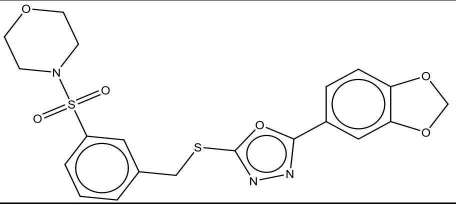	61.89±2.34
**152**	TCMDC-143536	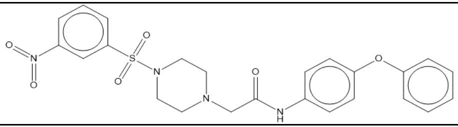	-2.95±1.70
**153**	TCMDC-143586	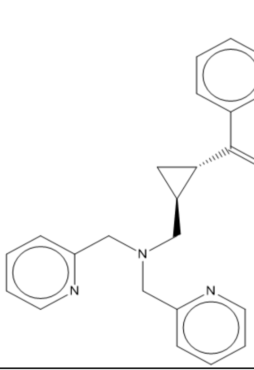	-5.50±2.15
**154**	TCMDC-143096	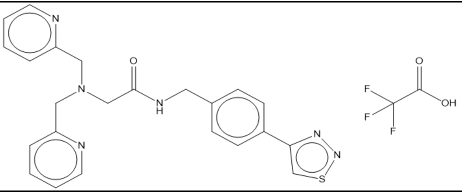	2.50±1.45
**155**	TCMDC-143119	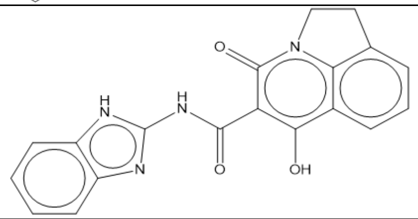	7.50±1.00
**156**	TCMDC-143139	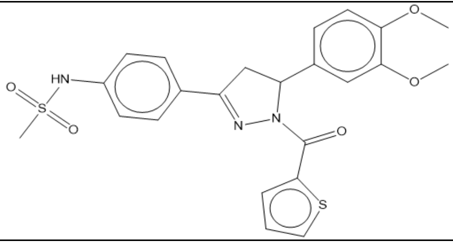	11.90±1.75
**157**	TCMDC-143557	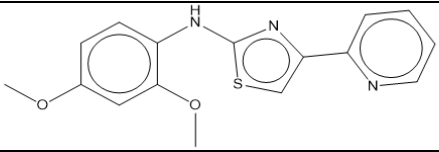	4.50±4.45

**158**	TCMDC-143170	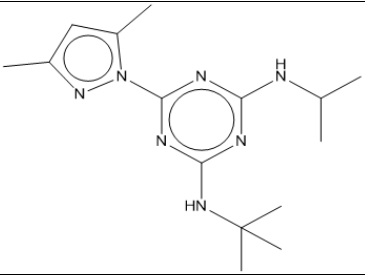	7.85±3.95
**159**	TCMDC-143306	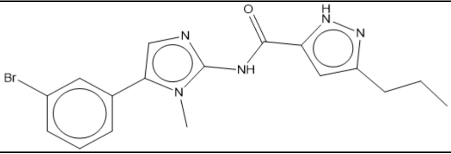	8.80±2.70
**160**	TCMDC-143345	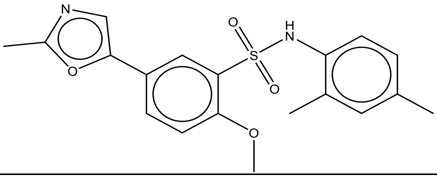	12.60±3.50
**161**	TCMDC-143315	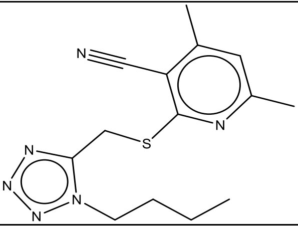	3.00±3.05
**162**	TCMDC-143532	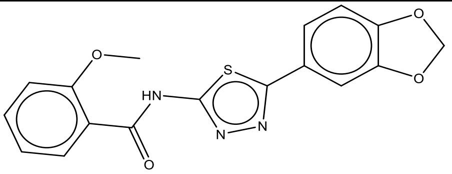	9.20±3.30
**163**	TCMDC-143591	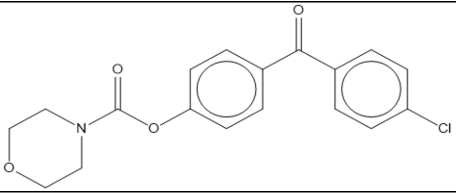	3.10±0.70
**164**	TCMDC-143573	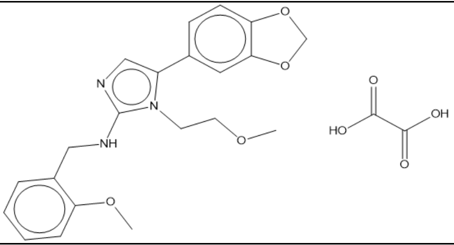	1.15±1.40
**165**	TCMDC-142704	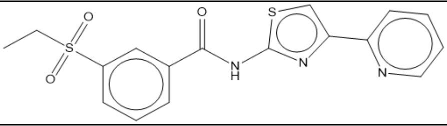	4.70±0.85

**166**	TCMDC-143174	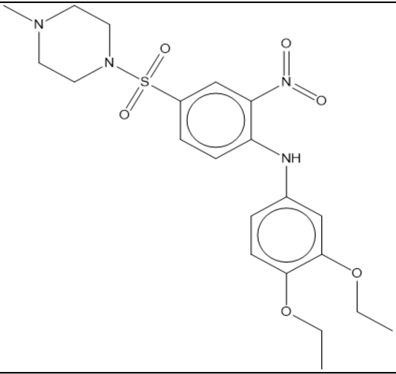	18.60±2.15
**167**	TCMDC-143237	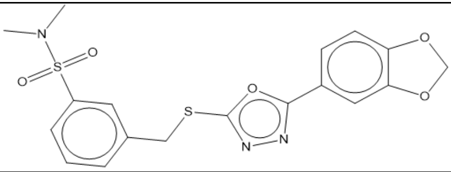	38.45±1.35
**168**	TCMDC-143252	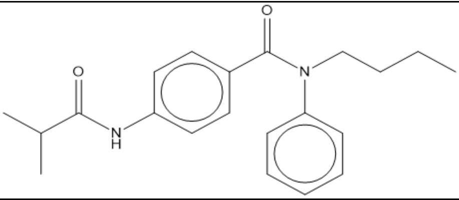	14.15±2.15
**169**	TCMDC-143236	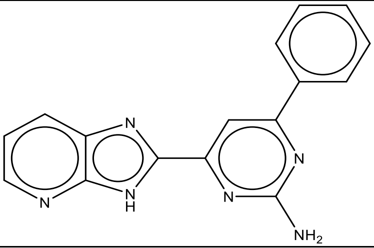	32.90±2.70
**170**	TCMDC-143344	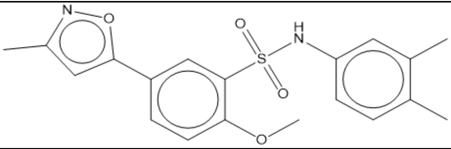	13.20±1.65
**171**	TCMDC-143639	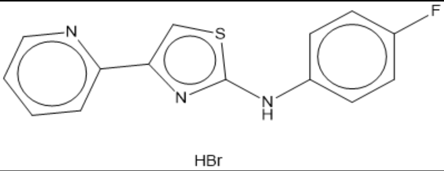	4.25±2.30
**172**	TCMDC-143523	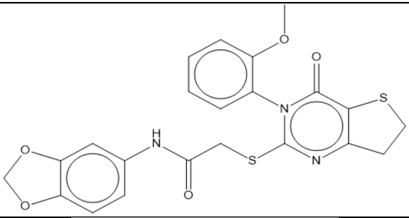	5.20±2.65
**173**	TCMDC-143538	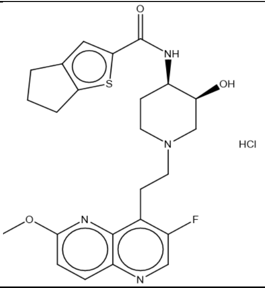	8.60±2.90

**174**	TCMDC-143574	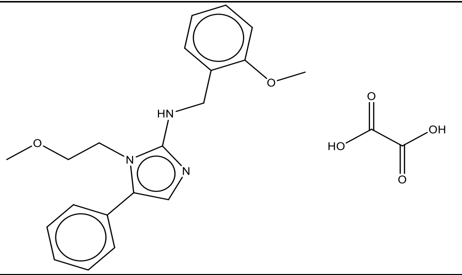	6.00±1.50
**175**	TCMDC-143101	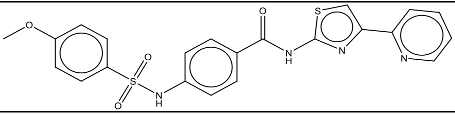	16.65±1.70
**176**	TCMDC-143165	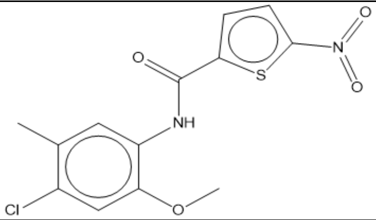	8.55±1.75
**177**	TCMDC-143218	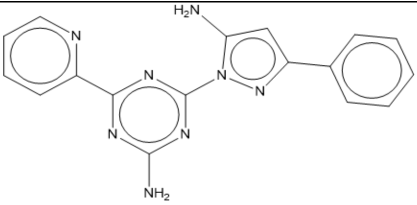	17.70±1.20
**178**	TCMDC-143255	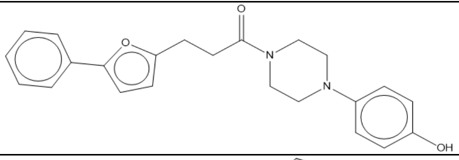	28.65±1.95
**179**	TCMDC-143327	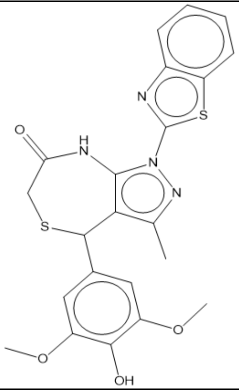	29.35±2.80
**180**	TCMDC-143358	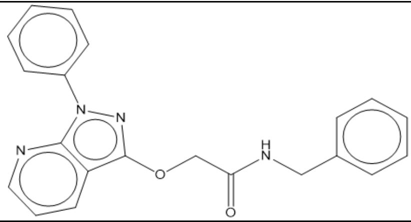	17.50±3.45
**181**	TCMDC-143517	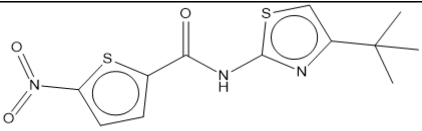	5.90±2.90

**182**	TCMDC-143518	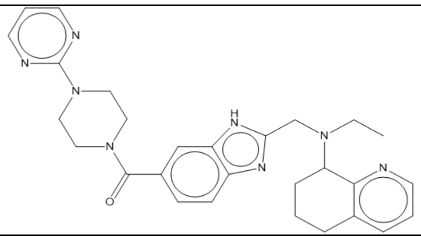	9.60±1.20
**183**	TCMDC-143514	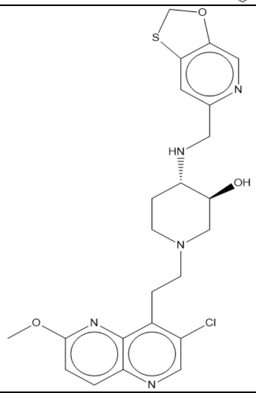	14.80±2.25
**184**	TCMDC-143098	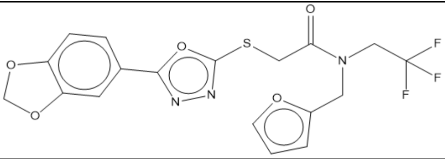	53.23±3.29
**185**	TCMDC-125387	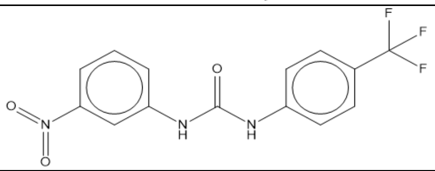	1.70±0.75
**186**	TCMDC-143166	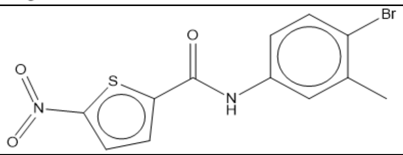	3.65±2.60
**187**	TCMDC-143181	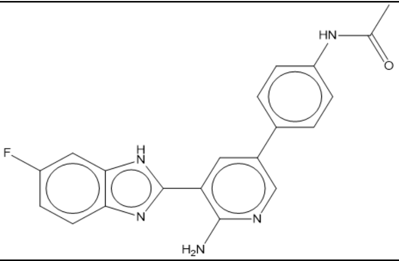	67.81±1.17
**188**	TCMDC-143287	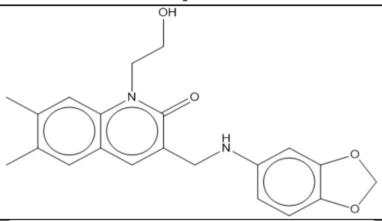	20.90±4.45
**189**	TCMDC-143348	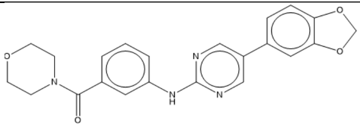	12.20±0.75

**190**	TCMDC-143508	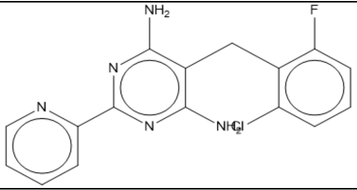	8.15±3.40
**191**	TCMDC-143531	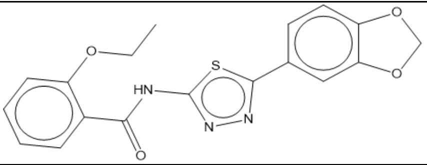	12.20±2.70
**192**	TCMDC-143568	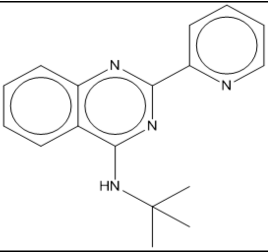	-2.40±2.70
**193**	TCMDC-143093	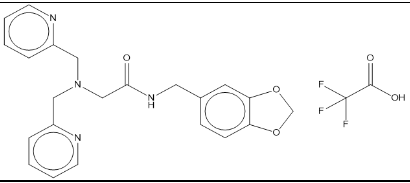	2.65±4.15
**194**	TCMDC-143566	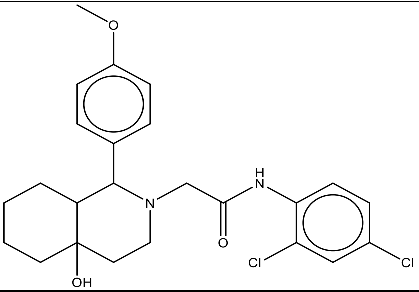	0.90±4.25
**195**	TCMDC-143140	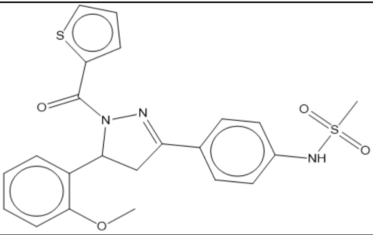	16.55±4.05
**196**	TCMDC-143188	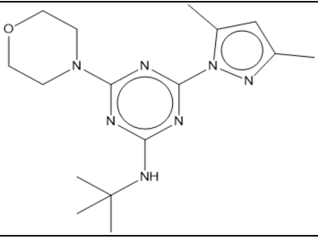	-2.80±6.35
**197**	TCMDC-143201	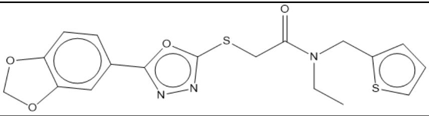	10.05±1.90
**198**	TCMDC-143268	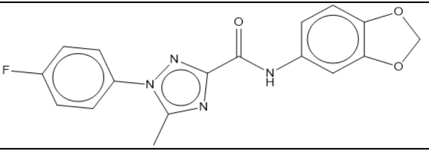	7.15±1.10

**199**	TCMDC-143355	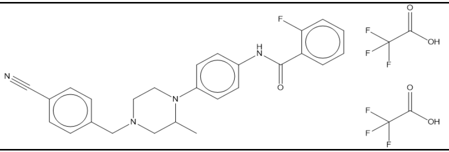	8.70±1.75
**200**	TCMDC-143383	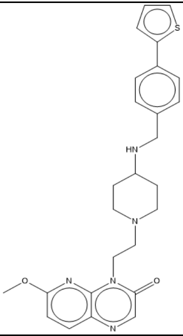	20.95±1.30
**201**	TCMDC-143522	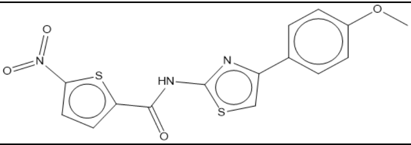	18.65±3.35
**202**	TCMDC-143570	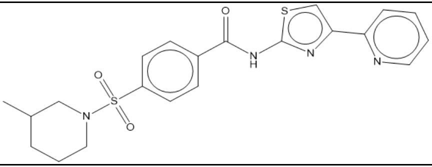	6.50±2.15
**203**	TCMDC-143090	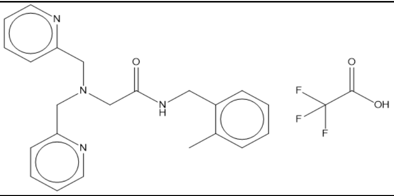	4.90±2.30
**204**	TCMDC-143117	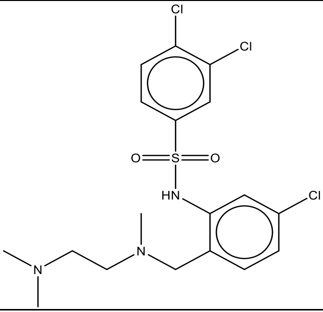	8.00±4.85
**205**	TCMDC-143184	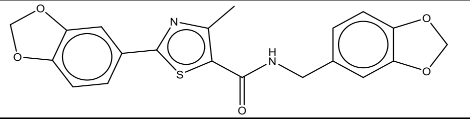	14.00±2.25
**206**	TCMDC-143202	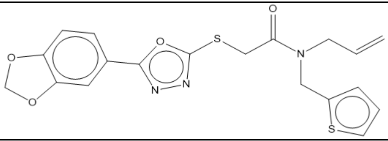	10.00±0.90
**207**	TCMDC-143271	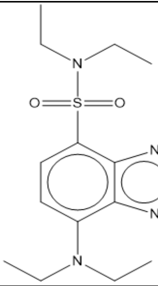	8.90±5.85

**208**	TCMDC-143259	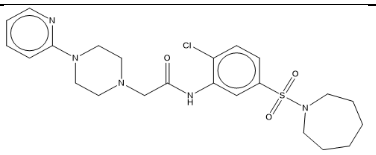	10.20±3.25
**209**	TCMDC-143340	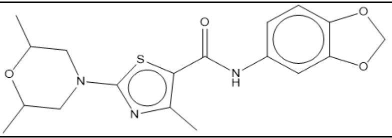	16.10±4.80
**210**	TCMDC-143618	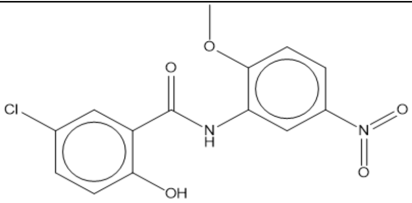	17.65±3.55

Table S3. LdGSK-3 inhibition for *N*-phenylpyrimidine-2-amines from the Leishbox.

Compound	Leishbox ID	Chemical Structure	%inhibition @10μM
**95**	TCMDC-143483	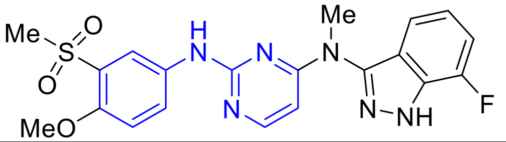	90.25±1.12
**74**	TCMDC-143451	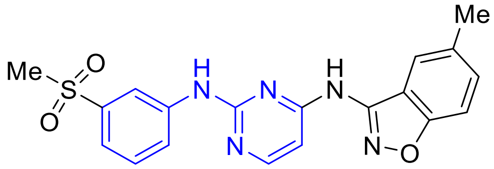	10.00±0.75
**124**	TCMDC-143391	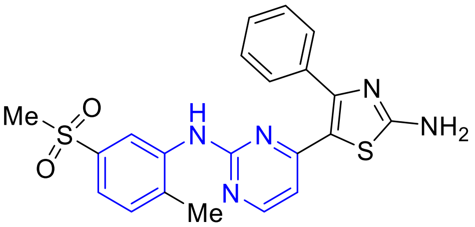	70.59±3.12
**140**	TCMDC-143367	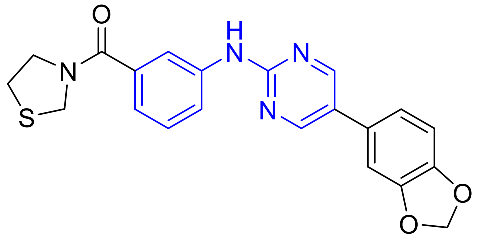	18.20±7.00
**67**	TCMDC-143349	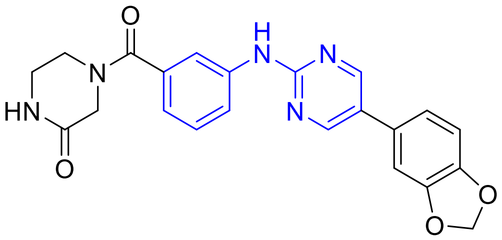	5.65±3.60
**189**	TCMDC-143348	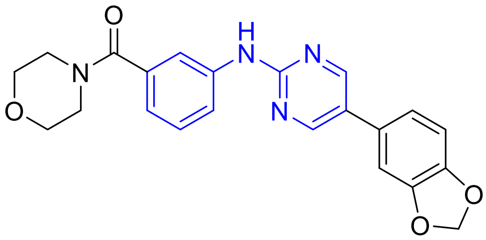	12.20±0.75
**119**	TCMDC-143281	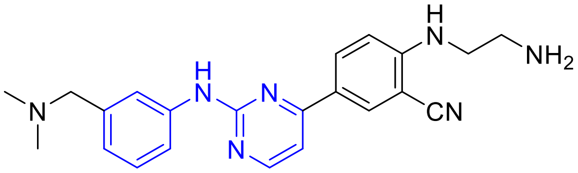	100.29±1.24
**128**	TCMDC-143280	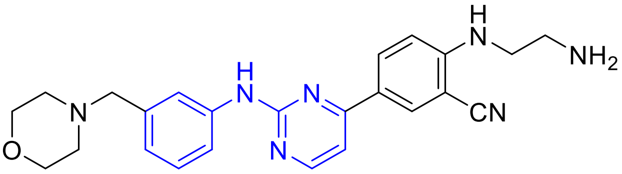	99.46±0.78

**100**	TCMDC-143249	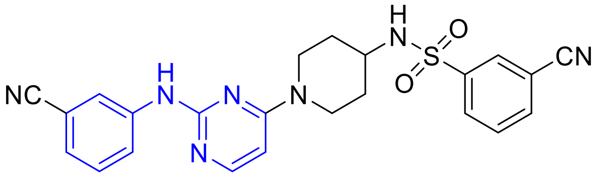	37.05±1.50
**73**	TCMDC-143246	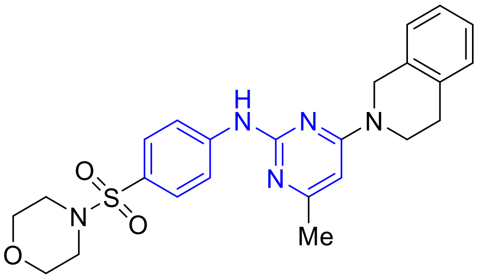	-3.05±0.35
**42**	TCMDC-143216	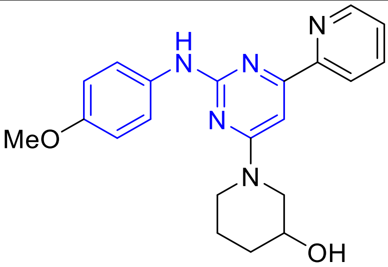	5.30±2.55
**48**	TCMDC-143215	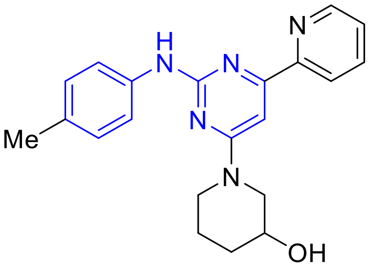	3.25±2.75
**99**	TCMDC-143214	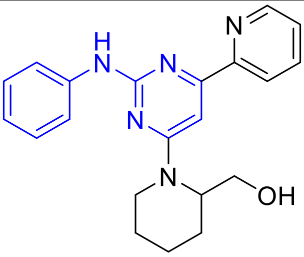	0.60±0.60
**45**	TCMDC-143213	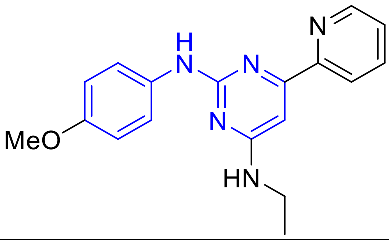	-2.50±5.50
**38**	TCMDC-143212	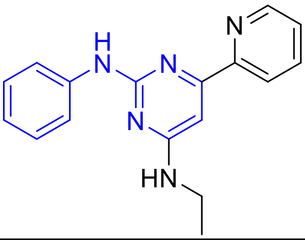	7.10±1.40

**34**	TCMDC-143211	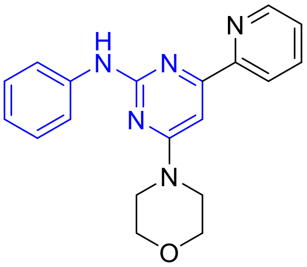	-2.00±2.70
**103**	TCMDC-143086	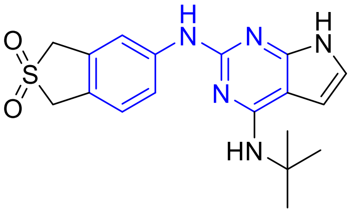	25.85±1.25

Table S4. LdGSK-3 inhibition for benzoimidazoles from the Leishbox

Compound	Leishbox ID	Chemical Structure	%inhibition @10μM
**40**	TCMDC-143554	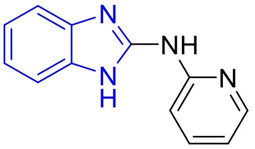	13.50±3.10
**91**	TCMDC-143524	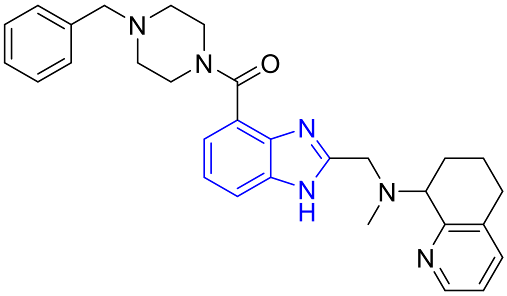	12.65±6.10
**182**	TCMDC-143518	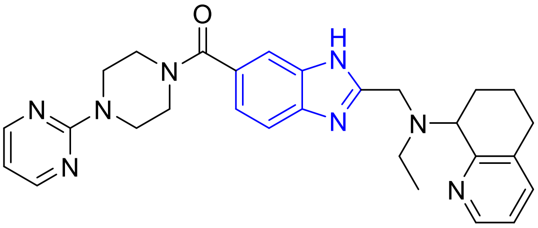	9.60±1.20
**28**	TCMDC-143512	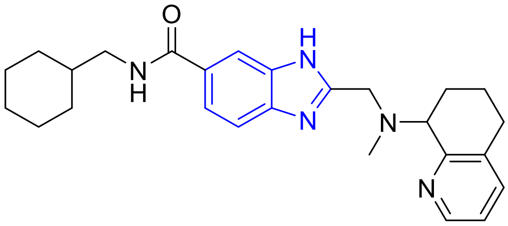	1.15±1.75
**85**	TCMDC-143503	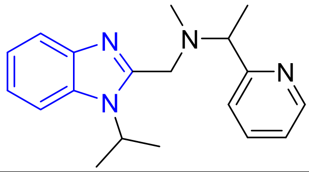	3.45±4.90
**114**	TCMDC-143478	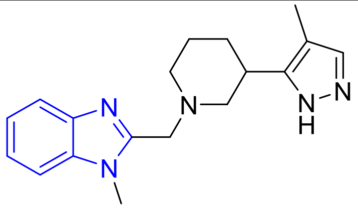	2.85±0.45
**120**	TCMDC-143429	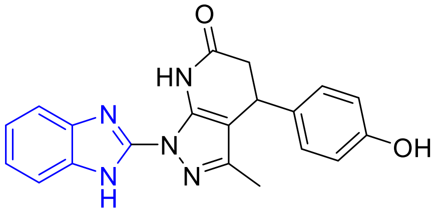	18.15±1.70

**71**	TCMDC-143396	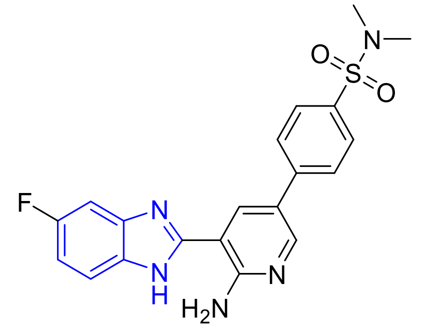	74.63±1.76
**53**	TCMDC-143350	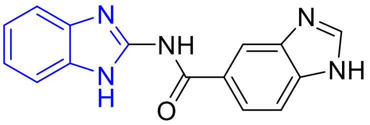	-1.60±1.50
**138**	TCMDC-143278	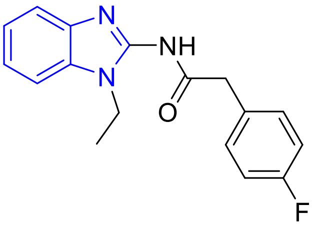	13.0±3.55
**70**	TCMDC-143277	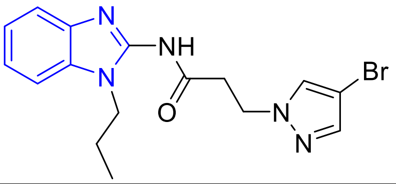	3.80±1.25
**80**	TCMDC-143261	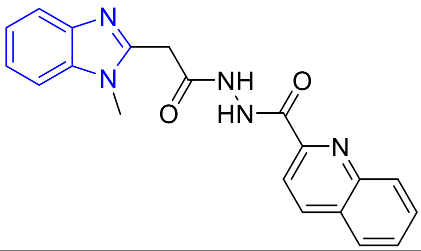	7.95±1.65
**187**	TCMDC-143181	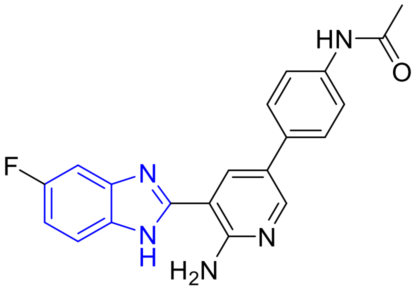	67.81±1.17
**79**	TCMDC-143168	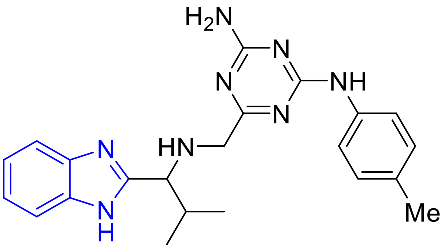	6.40±1.30
**155**	TCMDC-143119	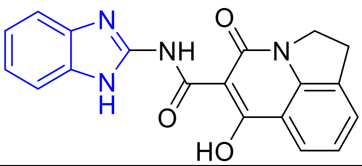	7.50±1.00

Table S5. LdGSK-3 inhibition for oxadiazole from the Leishbox

Compound	Leishbox ID	Chemical Structure	%inhibition @10µM
**125**	TCMDC-143594	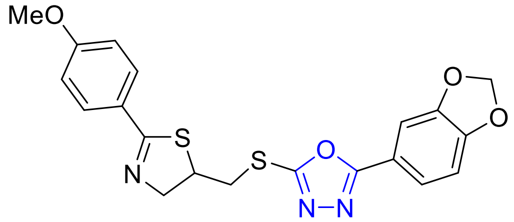	10.60±1.00
**167**	TCMDC-143237	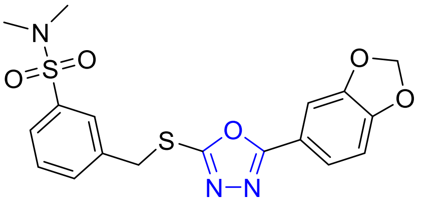	38.45±1.35
**206**	TCMDC-143202	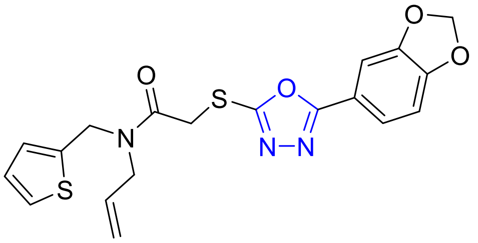	10±0.90
**197**	TCMDC-143201	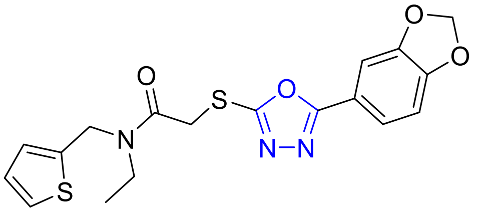	10.05±1.90
**184**	TCMDC-143098	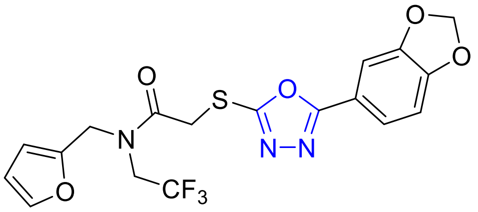	53.23±3.29
**151**	TCMDC-143197	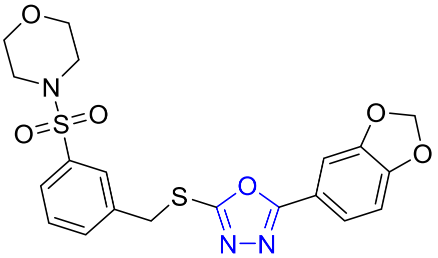	61.89±2.34

References

1. Martinez A, Alonso M, Castro A, Perez C, Moreno FJ. First non-atp competitive glycogen synthase kinase 3 beta (gsk-3beta) inhibitors: Thiadiazolidinones (tdzd) as potential drugs for the treatment of alzheimer's disease. J Med Chem 2002;45: 1292-1299.
2. Dominguez JM, Fuertes A, Orozco L, del Monte-Millan M, Delgado E, Medina M. Evidence for irreversible inhibition of glycogen synthase kinase-3beta by tideglusib. J Biol Chem 2012;287: 893-904.
3. Palomo V, Perez DI, Perez C, Morales-Garcia JA, Soteras I, Alonso-Gil S, Encinas A, Castro A, Campillo NE, Perez-Castillo A et al. 5-imino-1,2,4-thiadiazoles: First small molecules as substrate competitive inhibitors of glycogen synthase kinase 3. J Med Chem 2012;55: 1645-1661.
4. Conde S, Perez DI, Martinez A, Perez C, Moreno FJ. Thienyl and phenyl alpha-halomethyl ketones: New inhibitors of glycogen synthase kinase (gsk-3beta) from a library of compound searching. J Med Chem 2003;46: 4631-4633.
5. Perez DI, Conde S, Perez C, Gil C, Simon D, Wandosell F, Moreno FJ, Gelpi JL, Luque FJ, Martinez A. Thienylhalomethylketones: Irreversible glycogen synthase kinase 3 inhibitors as useful pharmacological tools. Bioorg Med Chem 2009;17: 6914-6925.
6. Palomo V, Soteras I, Perez DI, Perez C, Gil C, Campillo NE, Martinez A. Exploring the binding sites of glycogen synthase kinase 3. Identification and characterization of allosteric modulation cavities. J Med Chem 2011;54: 8461-8470.
7. Palomo V, Perez DI, Roca C, Anderson C, Rodriguez-Muela N, Perez C, Morales-Garcia JA, Reyes JA, Campillo NE, Perez-Castillo AM et al. Subtly modulating glycogen synthase kinase 3 beta: Allosteric inhibitor development and their potential for the treatment of chronic diseases. J Med Chem 2017;60: 4983-5001.
8. Perez DI, Palomo V, Perez C, Gil C, Dans PD, Luque FJ, Conde S, Martinez A. Switching reversibility to irreversibility in glycogen synthase kinase 3 inhibitors: Clues for specific design of new compounds. J Med Chem 2011;54: 4042-4056.
9. Perez-Domper P, Palomo V, Gradari S, Gil C, de Ceballos ML, Martinez A, Trejo JL. The gsk-3-inhibitor vp2.51 produces antidepressant effects associated with adult hippocampal neurogenesis. Neuropharmacology 2017;116: 174-187.
